# Combinatorial selective ER-phagy remodels the ER during neurogenesis

**DOI:** 10.1101/2023.06.26.546565

**Published:** 2023-06-26

**Authors:** Melissa J. Hoyer, Ian R. Smith, Julia C. Paoli, Yizhi Jiang, Joao A. Paulo, J. Wade Harper

**Affiliations:** 1 Department of Cell Biology, Harvard Medical School, Boston MA 02115; 2 Aligning Science Across Parkinson’s (ASAP) Collaborative Research Network, Chevy Chase, MD 20815, USA

## Abstract

The endoplasmic reticulum (ER) has a vast proteomic landscape to perform many diverse functions including protein and lipid synthesis, calcium ion flux, and inter-organelle communication. The ER proteome is remodeled in part through membrane-embedded receptors linking ER to degradative autophagy machinery (selective ER-phagy)^1,2^. A refined tubular ER network^3,4^ is formed in neurons within highly polarized dendrites and axons^5,6^. Autophagy-deficient neurons *in vivo* display axonal ER accumulation within synaptic ER boutons,^7^ and the ER-phagy receptor FAM134B has been genetically linked with human sensory and autonomic neuropathy^8,9^. However, mechanisms, including receptor selectivity, that define ER remodeling by autophagy in neurons are limited. Here, we combine a genetically tractable induced neuron (iNeuron) system for monitoring extensive ER remodeling during differentiation with proteomic and computational tools to create a quantitative landscape of ER proteome remodeling via selective autophagy. Through analysis of single and combinatorial ER-phagy receptor mutants, we delineate the extent to which each receptor contributes to both magnitude and selectivity of ER clearance via autophagy for individual ER protein cargos. We define specific subsets of ER curvature-shaping proteins or lumenal proteins as preferred clients for distinct receptors. Using spatial sensors and flux reporters, we demonstrate receptor-specific autophagic capture of ER in axons, which correlates with aberrant ER accumulation in axons of ER-phagy receptor or autophagy-deficient neurons. This molecular inventory of ER proteome remodeling and versatile genetic toolkit provides a quantitative framework for understanding contributions of individual ER-phagy receptors for reshaping ER during cell state transitions.

The ER network is shaped by the abundance of proteins that promote tubule and sheet-like membrane structures, which in turn tailors ER function in a cell type specific manner to optimize protein secretion, calcium storage, and lipid homestasis^3,4,10,11^. ER-phagy represents a central mechanism through which ER can be remodeled, or superfluous ER eliminated^2,12^. Several membrane-embedded ER proteins have been implicated in ER remodeling via ER-phagy in various cellular contexts with varying degrees of evidence. These ER-phagy receptors include single passing transmembrane (TM) containing proteins TEX264, CCPG1, SEC62 and reticulon-like hairpin domain (RHD) containing FAM134A, B, C, (also called RETREG2, 1, 3, respectively), Atlastin (ATL2), as well as RTN3L^8,13–20^. RHDs are thought to reside in the outer leaflet of the ER membrane to induce curvature^21–23^. All identified ER-phagy receptors contain cytosolic LC3-interaction region (LIR) motifs that bind to ATG8 proteins such as MAP1LC3B (also called LC3B) on the phagophore to promote ER capture^2^. Mechanistically, reticulon-type receptors are thought to cluster through their hairpin RHDs into highly curved nanoscale membrane domains that recruit autophagy machinery to emerging ER membrane “buds”, thereby nucleating phagophore formation^2,12,24–26^. Phagophore expansion and ultimate autophagosome closure around ER is coupled to scission of ER membrane at the bud neck through a poorly understood mechanism.

Given the complexity of ER-phagy receptors and the fact that most studies have involved either nutrient stress or receptor overexpression models, central unanswered questions in the field include when, where and how individual receptors are used to remodel ER during physiological changes in cell state and the extent to which individual ER proteins in unique cell states are susceptible to ER-phagic turnover. While ER protein accumulation has been observed in synaptic boutons of mouse primary hippocampal neurons from autophagy-deficient ATG5^−/−^ mice^7^, it has been suggested that ER proteome accumulation upon autophagy inhibition in neurons does not directly reflect selective ER-phagy receptor function but rather non-selective autophagy^7^. ER membranes serve as both a source of phospholipids for autophagosome expansion^27^ *and* are captured as cargo within a fully formed autophagosome via selective ER-phagy, as visualized by electron microscopy^14,15^. However, critical work has revealed that ER-embedded TMEM41/VMP1 proteins deliver phospholipids to ATG2, which then facilitates lipid transfer to ATG9 lipid scramblase in the nascent phagophore membrane without any incorporation of ER proteins into the phagophore membrane itself^27,28^. Thus, the process of ER-phagy receptor facilitated ER protein clearance is functionally and mechanistically distinct from the use of ER membranes as a source for phospholipids in phagophore expansion.

Here, we employ an in vitro neurogenesis system that recapitulates central autophagy-dependent features of ER remodeling^29^ to directly examine the role of ER-phagy receptors in ER remodeling, identify redundant and selective ER-phagic cargo for individual receptors, and demonstrate a role for multiple ER-phagy receptors in eliminating axonal ER. These data provide a quantitative proteomic landscape for ER remodeling in iNeurons and an experimental framework for elucidating how changes in cell state control the ER proteome via selective autophagy.

## Landscape of ER remodeling by autophagy during in vitro neurogenesis

**Extended Data Fig. 1.**
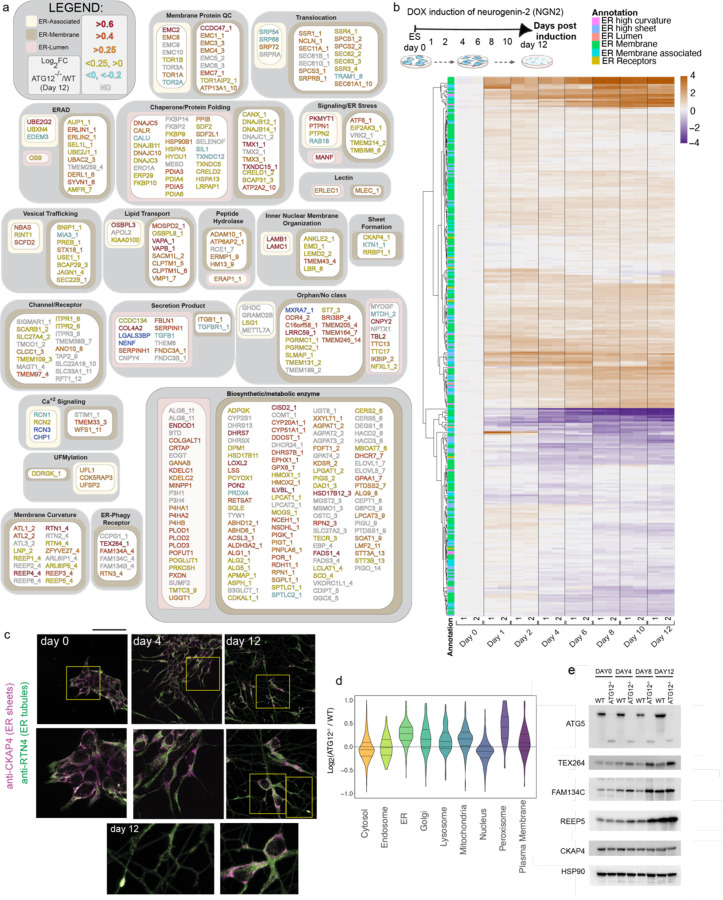
Landscape of ER remodeling via autophagy during hESC differentiation to iNeurons in vitro. **a**, Landscape of the ER proteome and the effect of autophagy on accumulation of individual proteins. The ER proteome (359 proteins, Supplementary Data Table 1) is organized into functional modules and protein attributes (involved in ER membrane curvature, ER-associated, ER-membrane, ER-Lumen or ER-phagy receptor) are indicated by the respective outline box color (see inset legend). For proteins with transmembrane segments, the number of segments are indicated after the protein name ( _1, _2, etc) based on data in Uniprot. The text of each protein name is colored based on day12 ATG12^−/−^ vs WT Log_2_FC (see inset legend). (Supplementary Data Table 3). **b**, Changes in the abundance of the ER proteome (267 detected proteins) during conversion of WT hESCs to iNeurons are shown in as heatmaps (Log_2_FC) at the indicated day of differentiation relative to hESCs. Data are from our previous analysis of iNeuron differentiation. Annotations of the type of ER protein are indicated by the relevant colors. **c**, hESCs were differentiated to iNeurons and stained with antibodies against CKAP4 enriched in ER sheets (magenta) and RTN4 enriched in ER-tubules (green) at day 0, 4 and 12 of differentiation. RTN4 staining is evident throughout neuronal processes. **d**, Violin plots for relative abundance of proteins located in the indicated organelles in ATG12^−/−^ versus WT day 12 iNeurons. **e**, Immunoblots of cell extracts from WT or ATG12 ^−/−^ hESCs for the indicated day of differentiation. Blots were probed with the indicated antibodies, with α-HSP90 employed as a loading control.

**Fig. 1.**
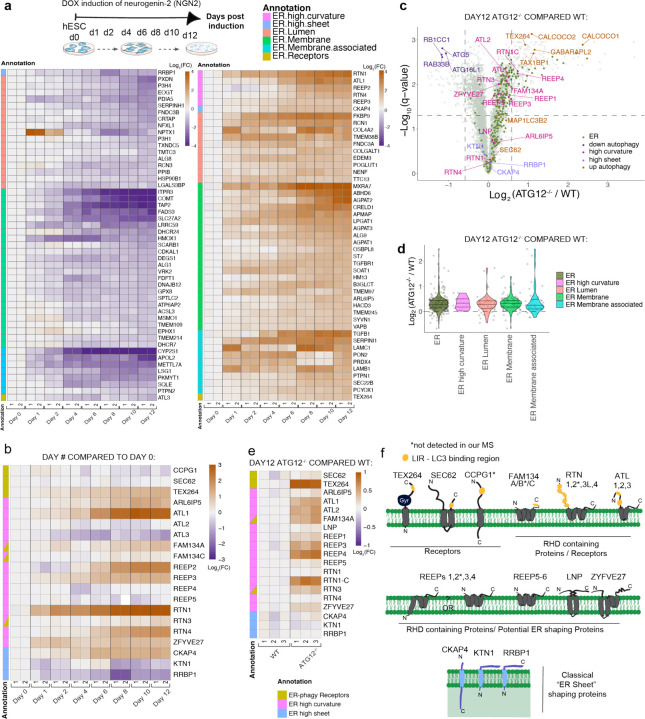
Landscape of ER remodeling via autophagy during hESC differentiation to iNeurons in vitro. **a**, Changes in abundance of the most highly remodeled ER proteins during conversion of WT hESCs to iNeurons are shown in heatmaps (Log_2_ Fold Change (FC) at indicated day of differentiation relative to hESCs). Data are from our previous analysis of iNeuron differentiation. Annotations depicting type of ER protein are indicated by the relevant colors. **b** Heat map (Log_2_FC) of ER shaping proteins specifically in differentiating iNeurons **c**, Volcano plot [-Log_10_ (q-value) versus Log_2_FC (ATG12^−/−^/WT)] of day 12 WT and ATG12^−/−^ iNeuron total proteomes displaying accumulation of autophagy-related and ER proteins (green dots) as a cohort. Each dot represents the average of triplicate TMT measurements. **d**, Violin plots for individual classes of ER proteins showing the relative increases in abundance in ATG12^−/−^ day 12 iNeurons compared with WT iNeurons. Each dot represents the average of triplicate TMT measurements. **e** Heat map (Log_2_FC) of ER shaping proteins specifically in day 12 WT versus ATG12^−/−^ iNeurons. **f**, Topology of ER shaping proteins and ER-phagy receptors within the ER membrane. Annotation color scheme for individual classes of ER proteins in **e** also applies to **b**.

The ER proteome is composed of ~350 proteins in four broad categories^30^: 1) membrane spanning proteins harboring one to as many as 14 TMs, 2) ER-associated proteins which are cytosolic but interact with ER-membrane, 3) lumenal ER proteins, and 4) ER tubule and sheet shaping proteins (Extended Data Fig. 1a and Supplementary Table 1). During a 12-day iNeuron differentiation, a cohort of ER proteins undergo dramatic changes in abundance, with examples in each ER annotation detected (Fig. 1a-b, Extended Data Fig. 1b, Supplementary Table 2)^29^. Proteins undergoing alterations in abundance include several enzymes involved in protein folding (e.g. FKBP9 increased; CRELD1 increased), ion regulation (e.g. RCN1 increased, TMEM38B increased), and collagen modification (COL4A2 increased, PXDN decreased, P3H4 decreased), or include secretion products that traffic through the ER lumen (Fig. 1a). Interestingly, among ER shaping proteins, RHD containing proteins RTN1, RTN4, and REEP2 all displayed substantial Log_2_ fold change increases (Fig. 1b, Extended Data Fig. 1b), consistent with the formation of extensive ER tubule networks within neuronal projections^31^. Indeed, immunofluorescence of RTN4 revealed extensive RTN4-positive projections in iNeurons, while CKAP4 (also called CLIMP63) was largely confined to the cell body (soma) (Extended Data Fig. 1c).

We next compared wildtype (WT) and ATG12^−/−^ day 12 iNeurons using Tandem Mass Tagging (TMT) proteomics (Fig. 1c, Supplemental Table 3). Consistent with previous results^29^, several autophagy cargo receptors (CALCOCO1, CALCOCO2, TAX1BP1, and the ATG8 protein GABARAPL2) accumulated in autophagy-deficient iNeurons, as did ER-phagy receptors TEX264 and FAM134A (Fig. 1c). Moreover, a cohort of ER proteins displayed increased abundance, as indicated by the rightward skew distribution in volcano plots of Log_2_ fold change (FC) values for ATG12^−/−^/WT proteomes and the complimentary violin plot displaying overall increase in ER abundance (Fig. 1c-d, Extended Data Fig. 1d). Strikingly, RHD proteins accumulate to the greatest degree (including REEP1–4, RTN1), while classical ER sheet proteins CKAP4 and RRBP1 were largely unchanged (Fig. 1d-f). Changes in protein abundance for TEX264, REEP5 and CKAP4 were verified by immunoblotting, as was increased abundance of FAM134C (not detected by proteomics in this experiment) (Extended Data Fig. 1e). Mapping the landscape of ER protein accumulation in ATG12 deletion iNeurons (Log_2_ FC from WT) revealed that, beyond ER curvature shaping proteins, specific ER proteins assigned to several other structural or functional categories accumulate during differentiation in the absence of autophagy, including lumenal and transmembrane segment-containing biosynthetic or metabolic proteins (Extended Data Fig. 1a).

## Aberrant axonal ER accumulation during neurogenesis without autophagy

**Extended Data Fig. 2.**
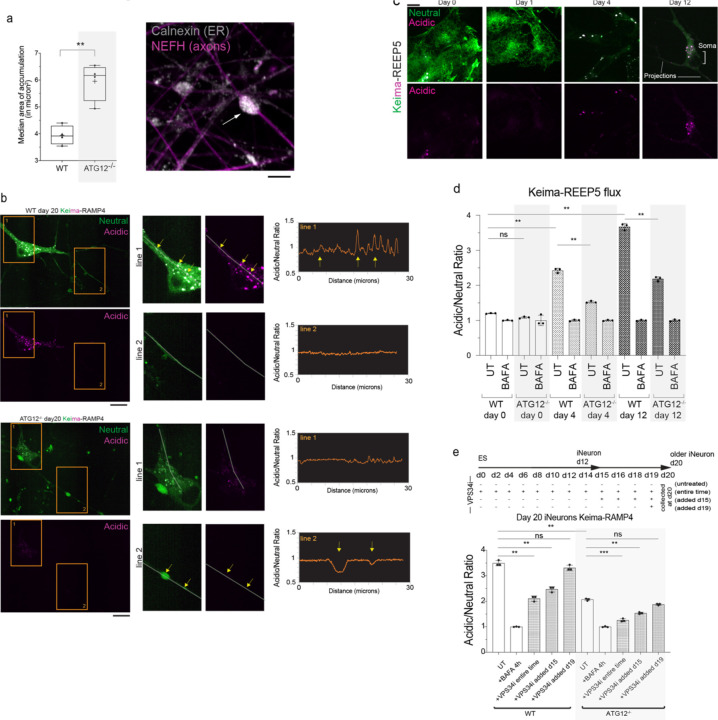
Autophagy-dependent clearance of ER in axons during iNeuron differentiation via ER-phagy receptors. **a**, ATG12^−/−^ day 12 iNeurons were immunostained with α-NEFH and α-Calnexin to identify aberrant ER structures. Scale bar, 5 microns. Right panel, min-to-max box-and-whiskers plot representing median areas of ER accumulations. The line is at the median. + labels the mean. Four points for each condition represent the resulting median area from four independent differentiations. **b**, Live cells expressing Keima-RAMP4 in WT and ATG12^−/−^ day 20 iNeurons were imaged. Scale bar, 10 microns. Insets show the results of acidic/neutral ratiometric line scan analysis for somata or axons of WT (top panels) or ATG12^−/−^ iNeurons. **c**, Induction of ER-phagic flux during iNeuron differentiation. hESCs expressing Keima-REEP5 were differentiated to iNeurons and Keima signal imaged at day 0, 1, 4 and 12. Scale bar, 10 microns. **d**, Ratiometric analysis of WT or ATG12^−/−^ Keima-REEP5 flux was measured by flow cytometry at day 0, 4 and 12 of differentiation. The ratio of acidic to neutral Keima fluorescence was normalized to samples treated with BAFA (100 nM) for 4 h prior to analysis. Each measurement reflects biological triplicate measurements. **e**, Ongoing ER-phagic flux in day 15 iNeurons. WT or ATG12^−/−^ hESCs were differentiated in the presence or absence of VPS34i as indicated in the scheme. In some cases, VPS34i was added at day 19 or day 15 and Keima flux analyzed by flow cytometry, as in panel **d**. Each measurement reflects biological triplicate measurements. *, p<0.05; **, p<0.01, ***, p<0.001.

**Fig. 2.**
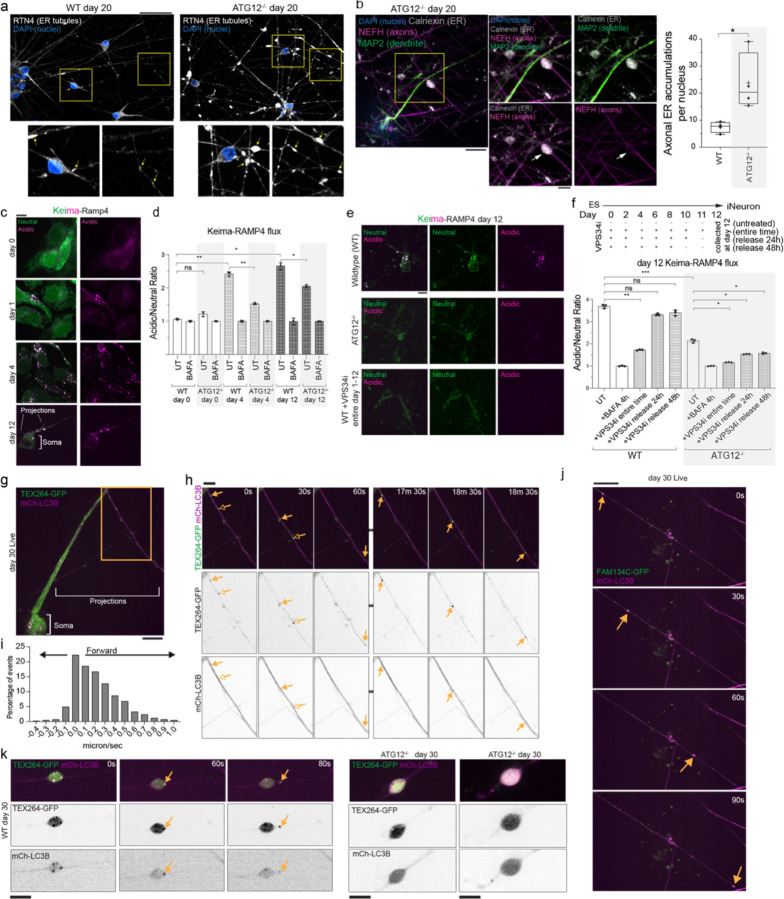
Autophagy-dependent clearance of ER in axons during iNeuron differentiation via ER-phagy receptors. **a**, WT or ATG12^−/−^ day 20 iNeurons immunostained with ER-tubule marker α-RTN4 (white) and with DAPI (nuclei, blue). Scale bar, 50 microns. **b**, Enlarged ER-positive structures in ATG12^−/−^ day 20 iNeurons revealed by immunostaining with α-Calnexin, ER (white); α-MAP2, dendrites (green); α-NEFH, axons (magenta); and DAPI, nuclei (blue). Scale bar, 10 microns. Right panel, min-to-max box-and-whiskers plot representing mean number of axonal ER accumulations per nucleus. Line marks median and + labels the mean. Points represent mean values from four independent differentiations. **c**, hESCs expressing Keima-RAMP4 were differentiated to iNeurons. Keima was imaged at day 0, 1, 4 and 12. Scale bar, 5 microns **d**, WT or ATG12^−/−^ Keima-RAMP4 flux was measured by flow cytometry at day 0, 4 and 12 of differentiation. The ratio of acidic to neutral Keima fluorescence was normalized to samples treated with BAFA (100 nM, 4 h). **e**, Reduced Keima-RAMP4 flux in ATG12^−/−^ iNeurons or upon VPS34 inhibitor, VPS34i (1 μM) treatment was measured as in **c**. Scale bar, 10 microns for **c** and **e**. **f**, WT or ATG12^−/−^ hESCs differentiated with or without VPS34i as indicated in the scheme. In some conditions, VPS34i was washed out at time indicated (24 or 48 h) prior to analysis. In **d** and **f**, each point represents one of three biological triplicate measurements. Error bars represent SD. For **b, d, f** *, p<0.05; **, p<0.01, ***, p<0.001. **g-h**, TEX264-GFP (green) and mCh-LC3B (magenta) day 30 iNeurons imaged live (panel **g**). Inset in panel **h** shows positions of mCh-LC3B/TEX264-GFP-positive puncta trafficking within an axon. Arrowheads indicate puncta positions over two indicated time sequences. Scale bars; 10 microns and 5 microns for **g** and **h**, respectively. **i**, Rate of 429 TEX264-GFP/mCh-LC3B-positive puncta movements, and the percentage of events at indicated speeds are binned in a histogram (events from three replicate differentiation experiments). **j**, As in **h**, but for FAM134C-GFP/mCh-LC3B-positive puncta. **k**, TEX264-GFP/mCh-LC3B-positive puncta are in dilated regions of WT iNeuron axons and traffic away, but puncta are not detected in ATG12^−/−^ iNeurons. Scale bars 10 microns.

We next examined ER morphology in WT or ATG12^−/−^ day 20 iNeurons using α-Calnexin or α-RTN4 as general or tubule-enriched markers for ER, respectively. We observed ER-positive accumulations that dilated the projections in autophagy-deficient cells. These ER accumulations were both larger and more numerous than those seen in WT iNeurons (Fig. 2a,b, Extended Data Fig. 2a). Immunofluorescent staining with α-NEFH (high molecular weight neurofilament-H) verified that the projections were axons (Fig 2b). Interestingly, α-NEFH-positive filaments formed a “cage-like” structure with multiple filaments encasing the ER (Fig 2b, inset). The median diameter of ER accumulations dilating the axons in ATG12^−/−^ iNeurons (in α-NEFH positive axon regions) was 6.17 micron^2^, while in WT iNeurons these were less abundant and consistently smaller (median diameter 3.92 micron^2^) (Fig. 2b, Extended Data Fig. 2a). These axonal regions filled with ER are reminiscent of previously observed boutons within mouse neurons lacking *Atg5* ^7^.

## ER-phagic flux during differentiation and in iNeurons

Curious as to the timing of ER accumulation in ATG12^−/−^ iNeurons, we next measured ER protein clearance to the lysosome (ER-phagic flux) at different stages of differentiation and in post-differentiated “established” iNeurons. We linked pH sensitive Keima to pan-ER (Keima-RAMP4) (Fig. 2c,d; Extended Data Fig. 2b) or to ER tubules (Keima-REEP5) (Extended Data Fig. 2c,d) and compared nonacidified Keima-ER throughout the ER network to acidified Keima-ER that had reached the low pH environment of lysosomes.^14,18,32^ Neither reporter underwent significant flux to lysosomes in ES cells, consistent with our previous data showing ER proteins do not accumulate in ATG12^−/−^ ES cells^29^. However, during differentiation, we observed a dramatic increase in acidic Keima signal (increased acidic/neutral ratio as defined in METHODS) for both ER reporters, with acidified puncta representing ER in lysosomes located primarily in the soma (Fig. 2c; Extended Data Fig. 2b,c). Parallel experiments using flow cytometry quantified the amount of ER flux to lysosomes upon differentiation using both reporters (Fig. 2d; Extended Data Fig. 2d). Acidic signal was normalized to cells treated with Bafilomycin A (BafA, 4h) which pharmacologically inhibits lysosomal acidification. This ER flux was substantially reduced in cells lacking ATG12, and residual flux was further eliminated by continuously adding small molecule VPS34 PI3 kinase inhibitor SAR405 (VPS34i) throughout the differentiation time course, which blocks phagophore initiation (Fig. 2e,f). Detectable flux in ATG12^−/−^ cells is consistent with the previous finding that loss of the ATG8 conjugation system does not fully block autophagosome formation^33^. Due to the long half-life of Keima in lysosomes^34^, detectable stable Keima within the lysosome over multiple days of differentiation was expected. Release from continuous VPS34 inhibition one or two days prior (at day 10 or 11) to neuron collection (at day 12) resulted in increased Keima flux to lysosomes that was comparable to flux in untreated cells; this increase was absent in cells lacking ATG12 (Fig. 2f). Finally, we examined whether ER-phagic flux was ongoing in established iNeurons. Keima flux measured in later stage day 20 neurons was reduced by adding VPS34i at day 15 of differentiation, as compared with untreated cells (Extended Data Fig. 2e). These results indicate that ER fluxes to lysosomes both throughout differentiation in a process that requires autophagy, and that autophagic ER flux is ongoing in established iNeurons.

## ER-phagy receptor capture by autophagosomes in axons and somata

Our findings left the localization of ER-phagy an apparent paradox: we observed acidic Keima-RAMP4 puncta within the soma (Extended Data Fig. 2b) but also detected dramatic ER accumulations within axons of ATG12^−/−^ iNeurons (Fig. 2a,b, Extended Data Fig. 2b). It is well known that autophagosomes originate in axons, fuse with lysosomes to form autophagolysosomes that acidify during retrograde trafficking en-route to the soma^35–38^. Thus, acidic Keima-RAMP4-positive puncta in the soma could reflect ER-phagy occurring locally in the soma or, alternatively, ER-phagic capture into autophagosomes within axons followed by retrograde transport to the soma.

**Extended Data Fig. 3.**
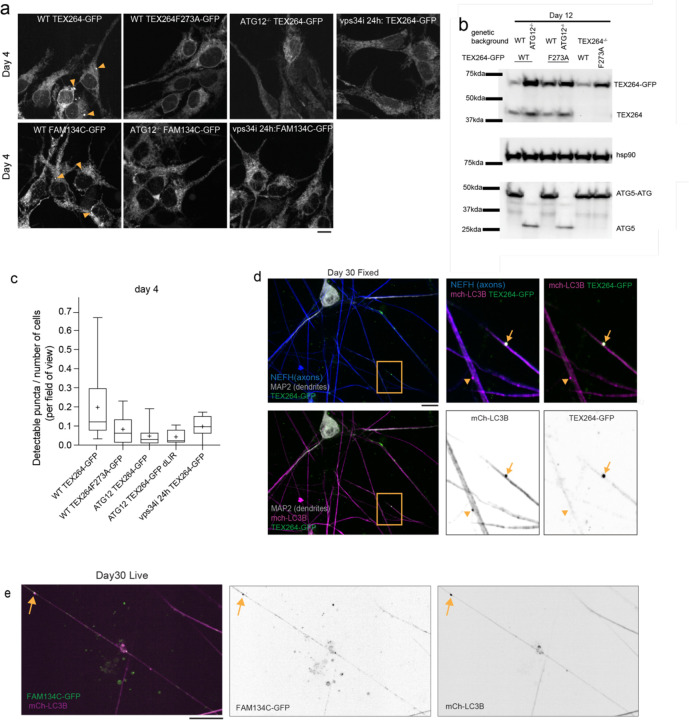
TEX264 and FAM134C puncta detection and tracking during iNeuron differentiation. **a-c**, TEX264-GFP, TEX264^F273A^-GFP, or FAM134C-GFP were expressed in WT or ATG12^−/−^ hESCs and cells imaged at day 4 of differentiation to iNeurons (panel **a**). In some experiments, VPS34i was added to WT cells for 24h prior to imaging. Arrowheads mark examples of ER-phagy receptor puncta In panel **b**, expression of TEX264-GFP was verified by immunoblotting of iNeuron extracts using α-HSP90 as a loading control. In panel **c**, the number of TEX264-GFP puncta was quantified in day 4 iNeurons. **d** Day 30 iNeurons expressing TEX264-GFP and mCh-LC3B were immunostained with α-MAP2 to detect dendrites (white) and α-NEFH to mark axons (blue) and processes imaged by confocal microscopy. Insets show TEX264-GFP/mCh-LC3B-positive puncta (arrows) or mCh-LC3B-positive but TEX264-GFP-negative puncta (arrowheads) in axons. **d** Separate channel images for FAM134C-GFP (green) and mCh-LC3B (magenta) for live example in main Fig. 2j. All scale bars, 10 microns.

To examine spatial aspects of ER-receptor capture, we expressed TEX264-GFP or FAM134C-GFP in iNeurons (Extended Data Fig. 3a-b). Previous studies (in non-neuronal cell lines) demonstrated that both TEX264 and FAM134 proteins can localize broadly throughout the ER network and can form puncta that become engulfed by autophagosomes^8,14^. We observed TEX264-GFP punctate structures (indicated by arrowheads) both in projections and in the soma (at day 4 of differentiation) that were rarely detected: 1) when TEX264’s LIR motif was mutated (F273A mutant), 2) in cells lacking ATG12, or 3) in cells treated with VPS34i (Extended Data Fig. 3a-c). These results suggest that ER-phagy receptor puncta formation in iNeurons was likely due to active ER-phagy as described previously in other cell systems that used starvation as a trigger for ER-phagy^8,14^.

To rigorously identify TEX264-GFP puncta in autophagic structures, we co-expressed mCherry-LC3B (mCh-LC3B). Co-staining with α-NEFH in fixed cells verified co-incidence of mCh-LC3B and TEX264-GFP in axons (Extended Data Fig. 3d). In live neurons, we tracked mCh-LC3B/TEX264-GFP-positive puncta movement in axons in live day 30 iNeurons. Numerous GFP-TEX264 puncta trafficked with mCh-LC3-positive structures (Fig. 2g,h, Movie 1). In each axon, autophagosomes enriched in TEX264 moved primarily unidirectionally (this predominant movement in one direction on the track is defined here as forward), but we also recorded stops and some backwards movements on these tracks (Fig. 2i). The median forward speed was 0.297 micron per second (Fig. 2i), which is like speeds previously reported for autophagosomes undergoing microtubule-dependent trafficking in axons of mouse primary neurons^39^. Similarly, dynamic FAM134C-GFP positive structures, trafficking with mCh-LC3B puncta, were also observed in day 30 iNeurons (Fig. 2j, Extended Data Fig. 3e, Movie 2), indicating that multiple ER-phagy receptors may be operating within projections.

Axonal dilations were detected in WT neurons, although less frequent and often smaller (Fig. 2a,b, Extended Data Fig. 2a). Interestingly, TEX264-GFP/mCh-LC3B-positive puncta were detected in these regions (Fig. 2k). Live cell imaging revealed exit of TEX264/LC3B-positive puncta out of these axonal dilations in WT neurons (Fig. 2k, Movie 3). TEX264-GFP was present in regions with dilated axonal ER in ATG12^−/−^ iNeurons, but TEX264-GFP/mCh-LC3B-positive puncta were not observed (Fig. 2k).Taken together, the finding that ER-phagy receptors are captured within autophagosomes trafficking out in axons, coupled with our finding that aberrant ER accumulations reside in axons of neurons that cannot perform autophagy, suggested a role for ER-phagy receptor-dependent clearance of ER in axonal processes.

## A genetic toolkit for combinatorial ER-phagy receptor analysis in iNeurons

**Extended Data Fig. 4.**
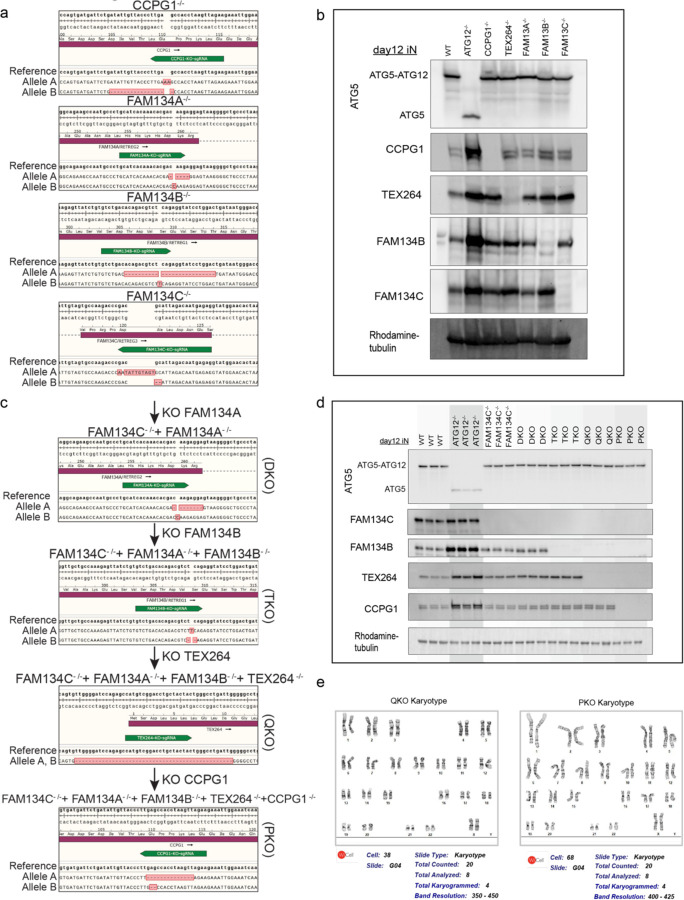
Generation of a genetic tool kit for functional analysis of ER-phagy receptors in iNeurons. **a**, MiSeq analysis of single ER-phagy receptor mutants in hESCs. The green highlights the target of the CRISPR gRNA. The sequence of the major MiSeq output is indicated for each allele. **b**, Immunoblot validation of targets knock-out clones at day 12. Cell extracts were subjected to immunoblotting with the indicated antibodies, employing a Rhodamine-labeled α-tubulin as loading controls. **c**, MiSeq analysis of combinatorial ER-phagy receptor mutants in hESCs, as performed for the single knockouts in a. **d,** Immunoblot validation of targets in combinatorial knock-out clones at day 12. Cell extracts were subjected to immunoblotting as in **b**. **e**, Karyotype analysis of QKO and PKO hESCs revealed no detectable alterations in chromosome number.

**Fig. 3.**
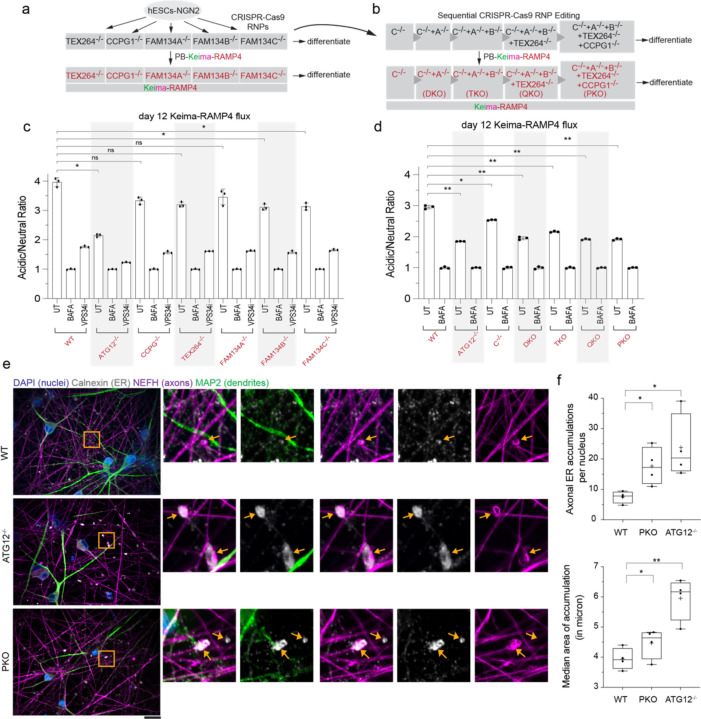
Combinatorial regulation of ER clearance via ER-phagy receptors during neurogenesis in vitro. **a-b**, A toolkit for analysis of ER-phagy receptors. hESCs were subjected to CRISPR-Cas9 gene editing to delete individual (panel **a**) or multiple (panel **b**) receptors. Keima-RAMP4 was expressed in each of the mutant hESCs, prior to analysis during differentiation. **c-d**, Ratiometric analysis of Keima-RAMP4 flux in the indicated WT or mutant hESCs was measured by flow cytometry at day 12 of differentiation. The ratio of acidic to neutral Keima fluorescence was normalized to samples treated with BAFA (100 nM) for 4 h. Each measurement reflects biological triplicate measurements. Error bars represent SD. **e-f**, PKO iNeurons accumulate aberrant ER structures, particularly in axons. WT or day 20 iNeurons of the indicated genotypes were immunostained with α-Calnexin (ER, white), α-MAP2 (dendrites, green), α-NEFH (axons, magenta), and with DAPI (nuclei, blue). Scale bar, 25 microns. Number of axonal ER accumulations/nucleus (panel **f, top**) or the median area of ER accumulation (panel **f, bottom**) are represented with min-to-max box-and-whiskers plots. Lines are at medians and “+” symbols designate the means. Four points shown for each WT or KO condition represent the measured values from four independent differentiations. For **c, d, f** *, p<0.05; **, p<0.01, ***, p<0.001.

To systematically explore contributions of individual ER-phagy receptors to ER remodeling during iNeuron differentiation^40^, we used gene editing to first create single knock-out hESCs for FAM134A, FAM134B, FAM134C, TEX264, and CCPG1, which were confirmed by sequence analysis and immunoblotting of hESC extracts (Fig. 3a, Extended Data Fig. 4, a,b). Multiple ER-phagy receptors accumulate in ATG12^−/−^ cells during differentiation and previous studies in non-neuronal cells indicate partial redundancy among ER-phagy receptors^15^, so we also sequentially edited FAM134C^−/−^ cells to create double, triple, quad, and penta receptor knockout lines: FAM134A/C^−/−^ (DKO), FAM134A/B/C^−/−^ (TKO), FAM134A/B/C/TEX264^−/−^ (QKO) and FAM134A/B/C/TEX264/CCPG1^−/−^ (PKO) (Fig. 3b, Extended Data Fig. 4c,d). Sequential deletion of ER-phagy receptors was verified by sequence analysis and immunoblotting; QKO and PKO mutants displayed normal karyotypes (Extended Data Fig. 4, c,d,e). Each mutant cell line was reconstituted with Keima-RAMP4 to measure ER-phagic flux to the lysosome (Fig. 3a,b).

## Combinatorial receptor control of ER-phagic flux in iNeurons

**Extended Data Fig. 5.**
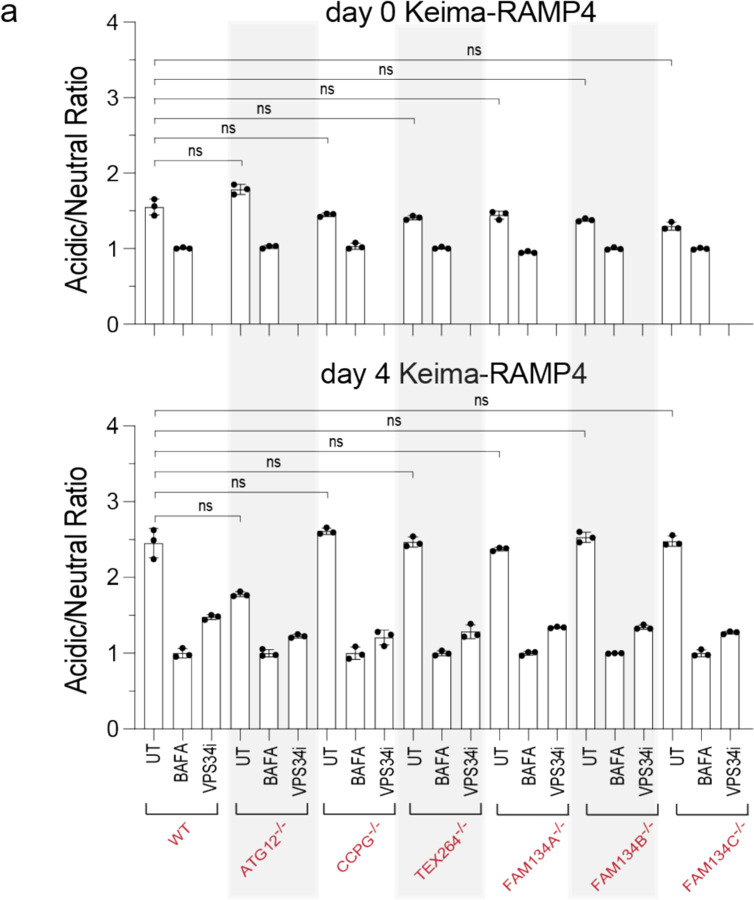
Combinatorial analysis of ER remodeling via ER-phagy receptors during neurogenesis in vitro. **a**, Ratiometric flow cytometry analysis of Keima-RAMP4 flux was measured in WT, ATG12^−/−^, or the indicated ER-phagy receptor knock-out cells at day 0 and 4 of differentiation. The ratio of acidic to neutral Keima fluorescence was normalized to samples treated with BAFA (100 nM) for 4 h prior to analysis, and where indicated, cells were cultured with VPS34i prior to analysis. Each measurement (represented by a point) reflects a biological triplicate sample. n.s. not significant. Error bars represent SD.

To directly examine individual receptor contribution to ER-phagy during differentiation, we measured Keima-RAMP4 ER clearance to the lysosome in receptor mutant cells at day 0, 4, or 12 of differentiation using flow cytometry (Fig. 3c, Extended Data Fig. 5a). As expected, Keima-RAMP4 flux increased from 2.5 to 4.0-fold in WT cells at day 4 and 12 of differentiation, which was substantially reduced in day 12 ATG12^−/−^ iNeurons (Fig. 3c, Extended Data Fig. 5a). In contrast, all single mutants displayed Keima-RAMP4 flux comparable to WT at day 4 and >80% of WT at day 12 (Fig. 3c, Extended Data Fig. 5a). However, upon elimination of multiple receptors, Keima-RAMP4 flux was further reduced, with the PKO mutant approaching a similar block in ER flux as ATG12^−/−^ cells at day 12 of differentiation (Fig. 3d). Thus, ER-phagy receptors exhibit redundancy in ER-phagic flux during iNeuron differentiation. Consistent with defective ER turnover, day 30 PKO iNeurons displayed more abnormally enlarged α-Calnexin marked ER structures in α-NEFH-positive axons (Fig. 3e). The number and size of these structures were intermediate between WT and ATG12^−/−^ iNeurons (Fig. 3f). These results demonstrate that ER-phagy receptors, as opposed to bulk autophagy, function to clear axonal ER during differentiation in vitro.

## Combinatorial receptor control of ER-proteome remodeling in iNeurons

**Extended Data Fig. 6.**
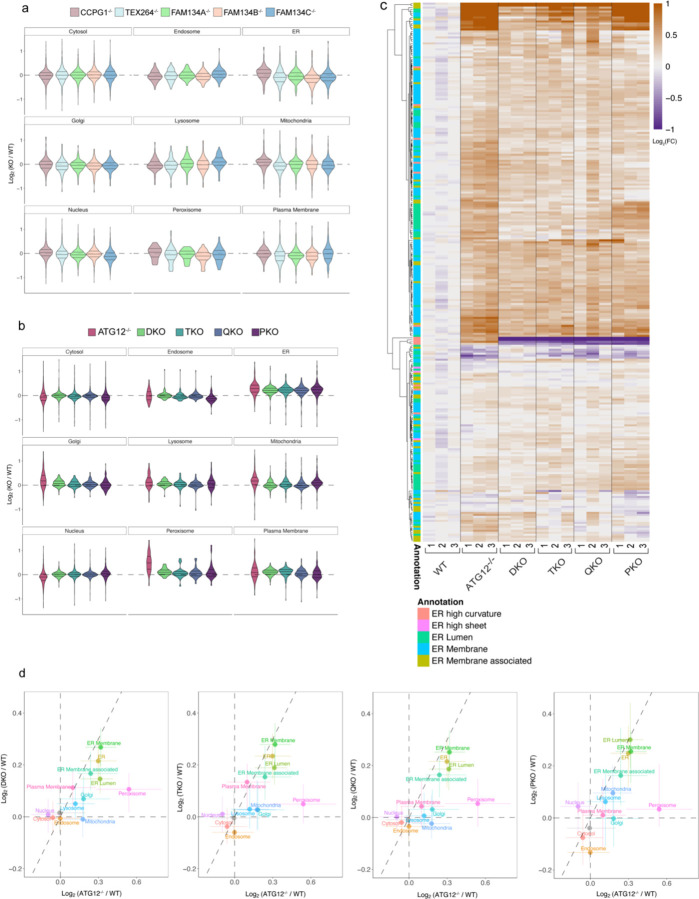
Combinatorial analysis of ER remodeling via ER-phagy receptors during neurogenesis in vitro. **a**, Violin plots for changes in individual organelle abundance in the indicated single ER-phagy knockout iNeurons (day 12). **b**, Violin plots for changes in individual organelle abundance in the indicated combinatorial ER-phagy knock-out iNeurons (day 12). **c**, Changes in the abundance (Log_2_FC) of the ER proteome (267 detected proteins) during conversion in ATG12^−/−^ or combinatorial ER-phagy receptor knockout iNeurons (day 12) are shown in as heatmaps. Annotations of the type of ER protein are indicated by the relevant colors. **d**, Correlation plots for changes in organelle abundance (Log_2_FC) comparing DKO, TKO, QKO and PKO individually with ATG12^−/−^ cells.

**Fig. 4.**
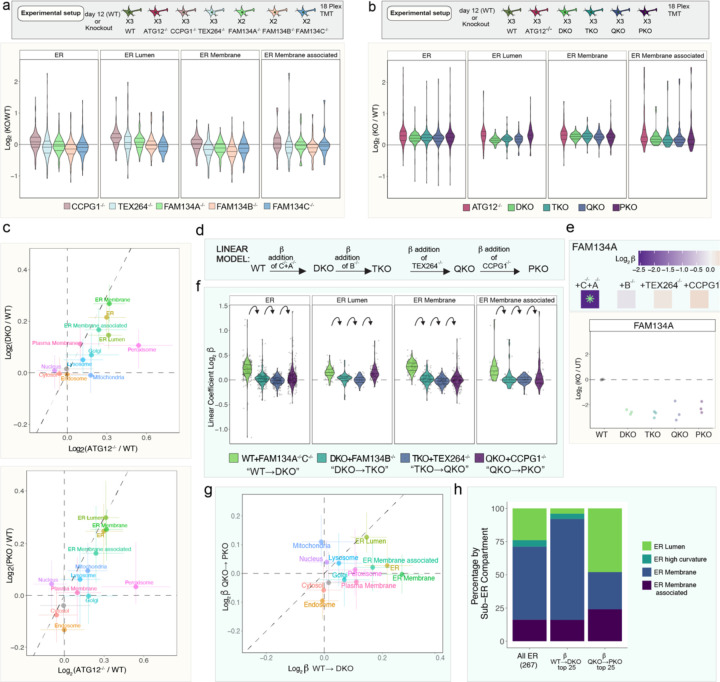
Selectivity of ER-phagy receptors in ER remodeling in iNeurons revealed by combinatorial multiplexed proteomics. **a**, Scheme depicting an 18-plex TMT experiment examining total proteomes of the indicated single ER-phagy receptor mutant day 12 iNeurons. Violin plots (lower panel) depicting Log_2_FC (mutant/WT) for the indicated classes of ER proteins in single mutant iNeurons (day 12) are shown in the lower plot. **b**, Scheme depicting an 18-plex TMT experiment examining total proteomes of the indicated combinatorial ER-phagy receptor mutant day 12 iNeurons. Violin plots (lower panel) depicting Log_2_FC (mutant/WT) for the indicated classes of ER proteins in combinatorial mutant iNeurons (day 12) are shown in the lower plot. **c**, Correlated accumulation of ER proteins in DKO or PKO iNeurons relative to ATG12^−/−^ iNeurons. Plots of Log_2_FC (ATG12^−/−^/WT) versus the indicated Log_2_FC (mutant/WT) for the indicated organelles. **d**, Application of a linear model to identify selective cargo for individual ER-phagy receptors via quantitative proteomics. In the linear model, a coefficient FC (β) is calculated for sequential loss of ER-phagy receptors starting from WT to DKO, then DKO to TKO, then TKO to QKO, then QKO to PKO. This analysis is distinct from traditional comparisons between each mutant and WT (lower panel). **e**, Top panels (β coefficient values) and lower panels (Log_2_FC) for FAM134A. Green asterisk in top panel indicated significant change (q-value <0.05) in β coefficient for that mutant. **e,** Violin plots depicting β coefficient FC for the indicated classes of ER proteins. **g**, Effect of WT to DKO versus QKO to PKO for individual organelles represented via β coefficient values. **h**, Top 25 accumulating ER proteins in WT to DKO and QKO to PKO and their respective ER compartment compared to the landscape of the whole ER.

While the results thus far revealed combinatorial control of the pan-ER Keima reporter flux and axonal ER clearance by ER-phagy receptors, the proteins subject to remodeling during differentiation and the specificity of ER clearance via individual receptors were unclear. To examine this question, we performed 18-plex TMT proteomics using single (Fig. 4a, Supplementary Table 4) and combinatorial (Fig. 4b, Supplementary Table 3) ER-phagy receptor mutants at day 12 of differentiation, with ATG12^−/−^ iNeurons as a control for autophagy-dependent stabilization. Abundance of organelles at the global level, including ER, was largely unaffected in single ER-phagy mutants, as indicated by violin plots for individual organelle proteomes (Fig. 4a, Extended Data Fig. 6a). In contrast and consistent with a more pronounced effect on Keima-RAMP4 flux and axonal ER accumulation, combinatorial mutants displayed an overall increase in ER protein abundance comparable to that seen in ATG12^−/−^ (Fig. 4b). The combinatorial mutants did not affect accumulation of other organelles in the same way as in ATG12^−/−^ iNeurons, which was consistent with a specific role in ER turnover (Extended Data Fig. 6b,c). Correlation plots with ATG12^−/−^ iNeurons revealed selective effects of ER-phagy receptor deletion on ER, with ER-membrane in particular accumulating similarly in ATG12^−/−^ and DKO iNeurons (Fig. 4c, Extended Data Fig. 6d). However, ATG12^−/−^ iNeurons had greater stabilization of lumenal ER proteins than DKO, TKO and QKO iNeurons, but additional removal of CCPG1 in PKO cells led to lumenal ER protein stabilization similar to that seen with ATG12^−/−^ iNeurons (Fig. 4d, Extended Data Fig. 6c,d).

**Extended Data Fig 7.**
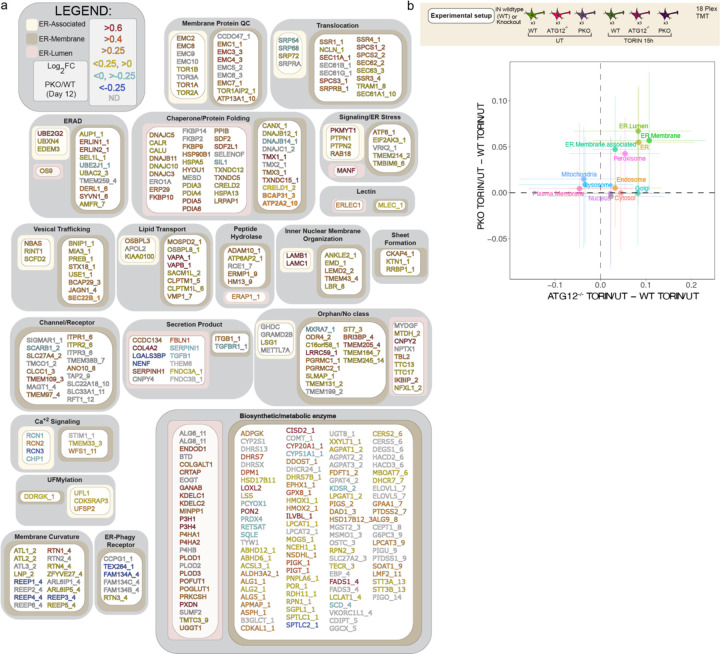
Landscape of the ER proteome in PKO iNeurons. **a**, Landscape of the ER proteome and the effect of deletion of five ER-phagy receptors (FAM134A/B/C, TEX263 and CCPG1) on accumulation of individual proteins. The ER proteome (359 proteins, Supplementary Data Table 1) is organized into functional modules and protein attributes (involved in ER membrane curvature, ER-associated, ER-membrane, ER-Lumen or ER-phagy receptor) are indicated by the respective outline box color (see inset legend). For proteins with transmembrane segments, the number of segments is indicated after the protein name (_1, _2, etc) based on data in Uniprot. The text of each protein name is colored based on day12 PKO vs WT Log_2_FC (see inset legend). (Supplementary Data Table 3). **b**, Modulation of iNeuron proteome in response to inhibition of MTOR with Torin1 (100 nM,15 h). Upper panel shows a schematic of the experimental set-up employing TMT based proteomics to quantity alterations in the proteome if WT or ATG12^−/−^, PKO iNeurons. Lower panel: Correlation plots comparing the effect of Torin1 on organelles of ATG12^−/−^ cells relative to WT cells and PKO cells relative to WT cells.

In cancer cell lines, MTOR inhibitor Torin1 induces a starvation-like response, leading to clearance of ER (among other organelles) and proteins via autophagy, but this clearance is blocked in autophagy deficient cells^41^.To further probe susceptibility of ER to selective turnover via general autophagy as compared with selective ER-phagy, we examined organelle and proteome abundance in iNeurons treated with Torin1 for 15 h. In ATG12^−/−^ iNeurons, organelle clearance was blunted when compared with WT iNeurons, which is consistent with autophagic turnover being blocked (Extended Data Fig. 7b, Supplementary Table 5). Consistent with findings that the PKO primarily affects only ER proteome remodeling, PKO iNeurons treated with Torin1 demonstrated a defect in clearance of ER proteins (similar to ATG12^−/−^), while other organelles were largely unaffected (in contrast to ATG12^−/−^) (Extended Data Fig. 7b, Supplementary Table 5).

Together, these data indicate that multiple ER-phagy receptors function to remodel the ER proteome during iNeuron differentiation. The landscape of ER protein accumulation in PKO iNeurons is displayed in Extended Data Fig. 7a.

## Quantitative modeling of ER proteome remodeling via ER-phagy

**Extended Data Fig. 8.**
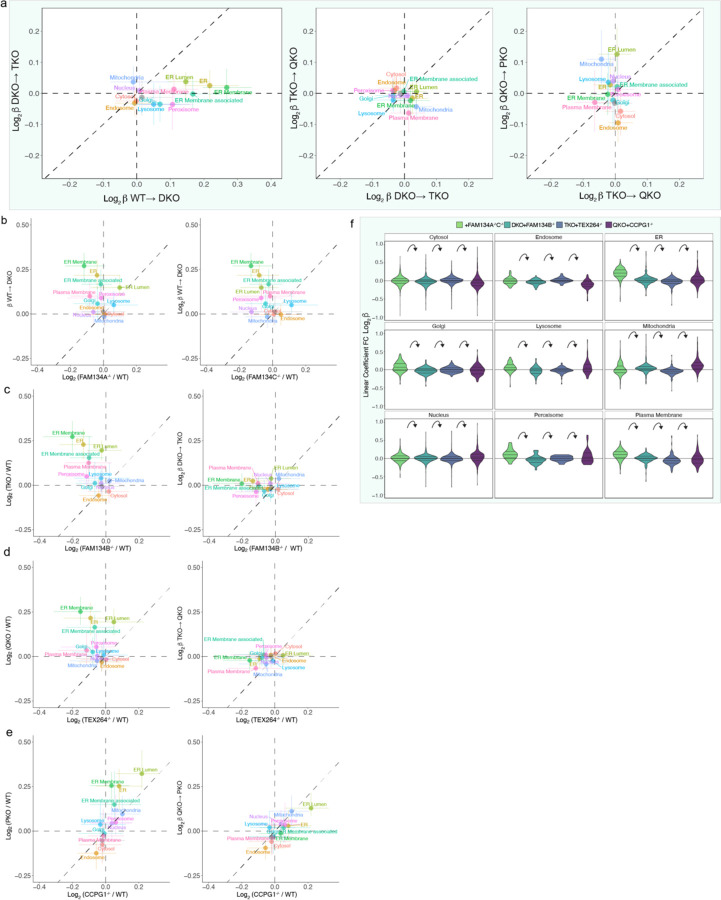
Application of a linear model for alterations in ER proteome abundance in sequential ER-phagy receptor knockout cells during iNeuron differentiation. **a**, Effect of sequential ER-phagy receptor deletion on the β coefficient values for individual organelles measured by quantitative proteomics in day 12 iNeurons. **b-e**, Correlation plots for the indicated β coefficient or Log_2_FC plots comparing organelle abundance for combinatorial or single ER-phagy deletion iNeurons. **f**, Violin plots reflecting changes in β coefficient values for individual organelles measured by quantitative proteomics in day 12 iNeurons. Curved arrows reflect sequential removal of the indicated ER-phagy receptor.

The behavior of various classes of ER proteins in single and combinatorial ER-phagy mutant iNeurons suggested the occurrence of both redundancy and selectivity in ER proteome remodeling during differentiation. Moreover, turnover of ER proteins by autophagy likely represents a continuum, with the fractional alteration in relative abundance dependent upon the extent to which ER proteins are present in the ER-phagic bud through either passive or active mechanisms. Thus, understanding selectivity across this continuum necessitates approaches that can identify potentially small alterations in the fractional abundance of the ER proteome. To address this challenge in the context of an ER-phagy receptor allelic series, we employed a linear model (see METHODS) (Fig. 4d). The resulting β coefficients reflect the extent to which individual protein abundance increases or decreases with each successive ER-phagy receptor deletion when compared with the preceding mutant in the allelic series [β^WT→DKO^; β^DKO→TKO^, β^TKO→QKO^, and β^QKO→PKO^, measuring the sequential effect of FAM134A/C, FAM134B, TEX264, and CCPG1 deletion, respectively] (Fig. 4d). To confirm the model, we used FAM134A protein levels as an example: β^WT→DKO^ was strongly negative (−2.5), consistent with its deletion in the DKO mutant compared to WT, but β coefficient values in subsequent deletions was near zero as expected since FAM134A remains deleted throughout the rest of the allelic series (Fig. 4e). Global analysis revealed an increase in mean β^WT→DKO^ coefficients for ER proteome (0.25), which was primarily reflected in alterations in the abundance of ER-membrane and ER-lumen proteins (Fig. 4f). In contrast, β^DKO→TKO^ and β^TKO→QKO^ coefficients reflecting the further deletion of FAM134B and TEX264, respectively, are near zero (Fig. 4f, Extended Data Fig. 8a). Collectively, this result indicates that FAM134B and TEX264 do not contribute appreciably to turnover of specific ER proteins in this system beyond those regulated by FAM134A and C (although see below for an exception). In contrast, the mean β^QKO→PKO^ coefficient increased by 0.11 for ER lumenal proteins (Fig. 4f,g, Extended Data Fig. 8a), indicating that CCPG1 and FAM134A/C independently control turnover of a set of lumenal proteins based on either the magnitude of abundance change or protein identity. The effect of CCPG1 on lumenal ER protein abundance is further demonstrated by organelle point plots comparing β^TKO→QKO^ and β^QKO→PKO^, with strong displacement of ER lumen off the diagonal (Extended Data Fig. 8a).

## ER-phagy receptor substrate specificity

**Extended Data Fig. 9.**
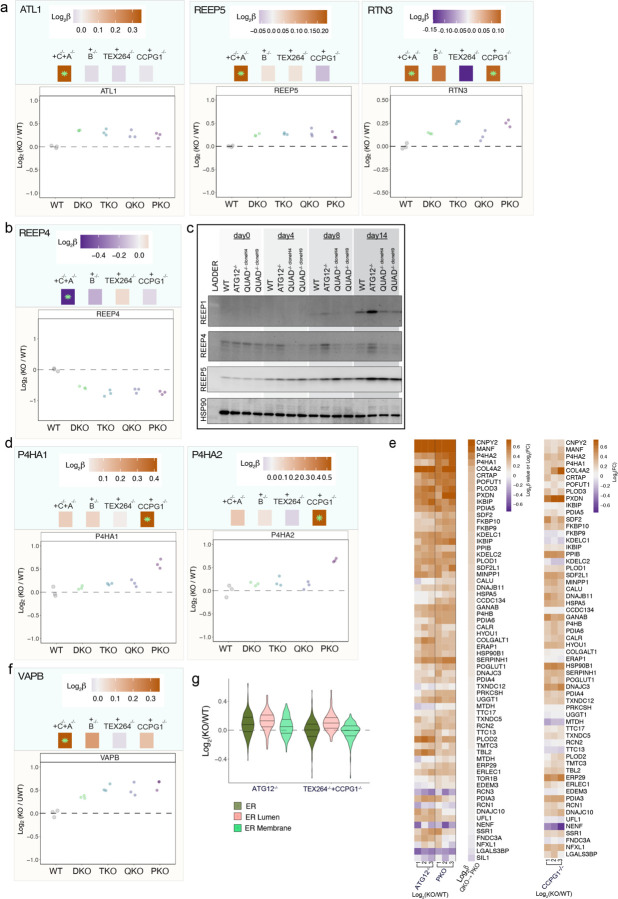
Differential regulation of ER membrane shaping proteins upon loss of ER-phagy receptors. **a-b**, Example ER shaping proteins that accumulate (**a**) or decrease (**b**) with additional ER-phagy receptor knockout **c**, Immunoblot of cell extracts isolated from the indicated time point during differentiation, using α-HSP90 as a loading control. **d**, ER lumenal proteins that accumulate with additional ER-phagy receptor knockout **e**, ER lumenal protein heatmaps reflecting the change in abundance (Log_2_FC) for deletion of ATG12 or PKO, reflecting β coefficient values for QKO to PKO, (left panel) or reflecting the change in abundance (Log_2_FC) for single deletion of CCPG1 (right panel) **f,** ER contact site protein that accumulates with additional ER-phagy receptor knockout. **g,** Violin plots from a TMT 18plex experiment comparing WT, ATG12^−/−^, and another ER-phagy receptor allelic combination (TEX264^−/−^ +CCPG1^−/−^), reflecting accumulation of the ER, ER lumen and ER membrane for Log2FC (ATG12^−/−^/WT) but only the ER luminal proteome accumulates for Log2FC (TEX264^−/−^ +CCPG1^−/−^ /WT). For single protein plots in **a, b, d, f** top panels are β coefficient values and lower panels are Log_2_FC; green asterisks in β coefficient for single protein heat maps indicate significant change (q-value < 0.05) in β coefficient.

**Fig. 5.**
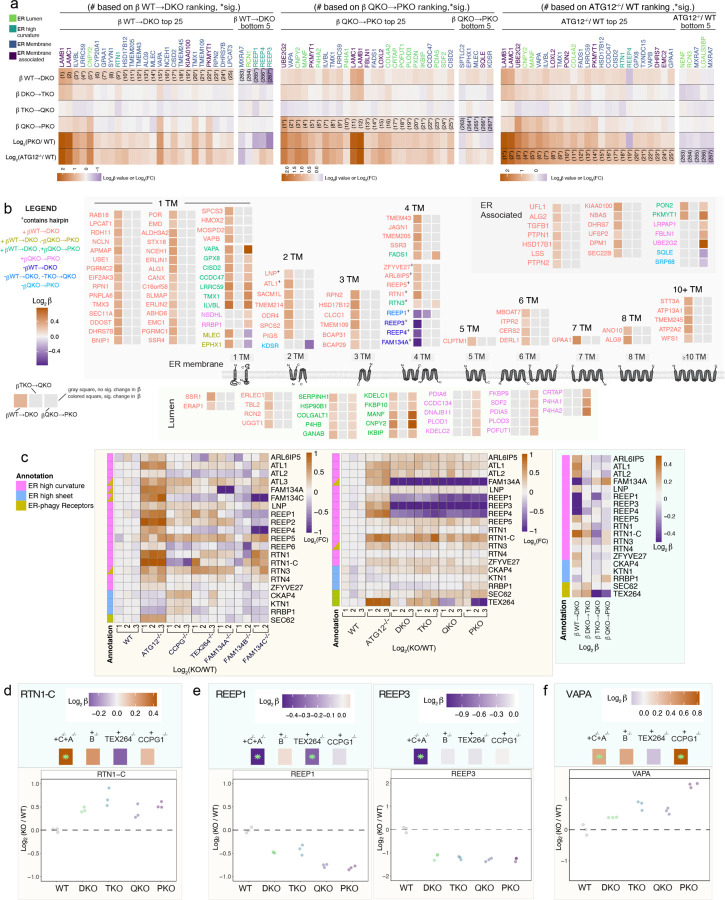
ER-phagy receptor remodeling of the ER proteome landscape and ER-phagy receptor cargo specificity during iNeuron differentiation. **a**, Top 25 accumulated and bottom 5 depleted ER proteins ranked on WT to DKO β coefficient values (left panel), QKO to PKO β coefficient values (middle panel), or on Log_2_FC (ATG12^−/−^/WT). **b**, The ER-associated, ER membrane, or ER lumenal distribution and predicted trans membrane character of ER proteins with significant β coefficient values (*, q-value <0.05) in WT to DKO (111 up, 4 down), TKO to QKO (1 down) and QKO to PKO (39 up, 5 down). Zero proteins were significant in DKO to TKO. Each protein name is colored based on if there is a significant change in these steps in the allelic series as according to the legend. The corresponding β coefficient value heatmap for each protein is colored in if there is a significant change and left blank if there is no significant change at that step in the allelic series (see legend). **c**, Heatmaps reflecting change in abundance (Log_2_FC) for the indicated ER shaping proteins in either single deletion (left panel), combinatorial deletion (middle panel) or reflecting the β coefficient values for combinatorial deletions. ATG12^−/−^ cells were analyzed together with ER-phagy mutants. Top panels (β coefficient values) and lower panels (Log_2_FC) for single proteins including RTN1-C (example ER shaping protein that accumulates), REEP1 and REEP3 (ER shaping proteins that decrease) or VAPA (ER membrane protein that forms contact sites with other organelles). Green asterisk in top panel indicates significant change (*, q-value <0.05) in β coefficient for that mutant.

To directly uncover substrate selectivity of ER-phagy receptors, we first explored the top 25 ranked proteins with positive β coefficients for both β^WT→DKO^ and β^QKO→PKO^. When compared with all ER proteins, those with positive β^WT→DKO^ coefficients were particularly enriched in ER membrane proteins while in contrast, proteins with positive β^QKO→PKO^ coefficients were enriched in lumenal proteins (Fig 4h). Remarkably, Log_2_FC values for these same individual proteins were also elevated in ATG12^−/−^ iNeurons (Fig. 5a), indicating that, in terms of the ER proteome, the PKO mutant closely approximates the biochemical phenotype of ATG12 deficiency. Globally, we identified 84 membrane proteins with significantly (q-value <0.05) positive or negative β^WT→DKO^ coefficients, which were distributed across multiple functional categories and contained varying numbers of TM segments (Fig. 5b and Supplementary Table 3). Given that ATG12 deficiency strongly affects ER shaping proteins with RHDs (Fig. 1e), we first examined this class of proteins in our allelic ER-phagy receptor deletion series. A subset of ER curvature proteins was significantly altered in β^WT→DKO^ (Fig. 5a-c, Extended Data Fig. 9a). In particular, RTN1-C accumulated with a substantial fold change (Log_2_FC=0.44 in DKO), which also accumulated in ATG12^−/−^ iNeurons (Log_2_FC=0.74) (Fig. 5a-d). Second, a distinct set of RHD proteins (REEP1, REEP3, REEP4) decrease in abundance, and display negative β coefficients for DKO (Fig. 5a-c,e and Extended Data Fig. 9b). REEP1 also further decreases upon deletion of TEX264, as indicated by a significant negative β-coefficient and Log_2_FC (Fig. 5c,e). Since members of the RHD protein family (e.g. REEP1) are strongly upregulated during iNeuron differentiation (Fig. 1a-d, Extended Data Fig. 9c), alterations in abundance across the REEP family indicate distinct pathways for controlling ER shape remodelling for neurons specifically via ER-phagy. Whereas the collective ER proteome did not increase for the single FAM134C deletion, abundance alterations for ER-shaping proteins specifically were observed with just the single deletion (Fig. 5c), indicating that FAM134C likely contributes substantially to differential regulation of shaping proteins during neurogenesis (Fig. 5c). Interestingly, ATG12^−/−^ iNeurons display increases in abundance for all REEP proteins, indicating that a broad block to autophagy can mask otherwise distinct proteome remodeling events relevant to an individual ER-phagy receptor (Fig. 5c).

The ER lumenal compartment is primarily responsible for folding and modification of secretory and membrane proteins, but proteins in this compartment have also been reported to undergo autophagic trafficking^13,42,43^. We identified two major patterns of ER lumenal protein abundance changes, reflected in β^WT→DKO^ and β^QKO→PKO^ coefficients. In total, 25 ER lumen proteins (primarily lacking a TM) were stabilized in the DKO mutant, and a subset of these (10) were further stabilized in PKO mutants (Fig. 5b and Extended Data Fig. 9d and Supplementary Table 3). In contrast, a distinct cohort of ER lumen proteins (16) was stabilized specifically in the PKO mutant with no significant effect observed with DKO, TKO, or QKO mutants (e.g. P4HA1 and P4HA2) (Fig. 5a,b and Supplementary Table 3), and Log_2_FC for these lumenal proteins were also stabilized in ATG12^−/−^ iNeurons (Extended Data Fig. 9e). These findings suggest both redundant and specific lumenal cargo for FAM134A/C and CCPG1 receptors. We also found it compelling that deletion of CCPG1 alone resulted in increased abundance of a subset of lumenal proteins with significant similarities to that seen with the PKO mutant (Extended Data Fig. 8e,9e). An independent proteomic analysis of iNeurons representing another allelic combination of ER-phagy receptors, a TEX264^−/−^/CCPG1^−/−^ double mutant, compared to ATG12^−/−^ and WT iNeurons independently verified selective ER lumenal protein clearance via CCPG1 (Extended Data Fig. 9g and Supplemental Table 6).

Intriguingly, the single TM segment proteins VAPA and VAPB, which mediate contact site interactions between ER and a number of other organelles, including mitochondria, via an interaction with VPS13 and other lipid transfer proteins^44^, have a positive β coefficient in DKO and/or PKO mutants, indicating that VAPs undergo multiple modes of ER-phagic turnover (Fig. 5f and Extended Data Fig. 9f). VAPA abundance was also increased ATG5^−/−^ cerebellar granule neurons in culture^7^. In parallel, β coefficient correlation plots for organelles reveals selective accumulation of mitochondria as a result of CCPG1 deletion (Extended Data Fig. 8e,f). These findings open further study into how ER-phagy mechanisms are regulating ER architecture to facilitate functions like maintaining robust yet dynamic contact sites with other organelles.

## DISCUSSION

Previous studies indicated that loss of autophagy pathways in mice or ES cells leads to increased accumulation of ER proteins^7,29^, but the extent to which this reflects non-specific macroautophagy or selective ER-phagy was unknown. Indeed, RNAi mediated suppression of individual ER-phagy receptors failed to promote axonal ER accumulation in cultured mouse neurons, leading to the suggestion that ER-phagy may be a constitutive process linked with autophagosome formation in distal axons^7^. The use of a genetically trackable iNeuron system, which displays a dramatic accumulation of axonal ER in the absence of a functional autophagy system^29^, has allowed us to examine roles for multiple ER-phagy receptors during ER remodeling associated with neurogenesis.

Through analysis of FAM134C and TEX264 trafficking in iNeurons, we found that these ER-phagy receptors are mobilized into LC3B-positive vesicles that traffic in axons. Previous studies demonstrated that FAM134 and TEX264 are concentrated into the same ER structures that are captured during ER-phagy while CCPG1 also undergoes ER-phagy but forms distinct ER-cargo domains^14^. Current models indicate that ER serves as a source of lipids for phagophore formation but that ER membrane proteins themselves are not incorporated into autophagosomal membranes^27,28^. Thus, we conclude that FAM134C and TEX264-positive puncta reflect ER-phagy rather than the process of autophagosome biogenesis as previously observed in distal axons^39^. Consistent with this, we readily observed LC3-positive autophagosomes that traffic on microtubules but lack detectable TEX264 or FAM134C proteins, indicative of distinct cargo (Extended Data Fig. 3d).

Generation of single and combinatorial ER-phagy receptor knockouts complimented with general ER flux measurements and quantitative proteomics revealed that selective ER-phagy mechanisms clear ER proteins during iNeuron differentiation. Mutation of FAM134A and C was necessary to produce a global increase in the ER proteome, with the transmembrane ER proteome featured prominently among the most stabilized proteins. In contrast, deleting CCPG1 in different allelic backgrounds revealed CCPG1’s primary role in clearing lumenal proteins. Unlike FAM134 family members and TEX264, CCPG1 contains a lumenal domain that has been suggested to associate with lumenal autophagy substrates^13,42,45^. Our proteomic analysis validates previously reported CCPG1 cargo (e.g. P3H4)^42^ and provides additional candidates for further analysis. Although our data point to a prominent role of FAM134A and C in ER-phagy in our allelic series with little further impact on FAM134B and TEX264, it is possible that these proteins have a similar overlapping substrate specificity that is masked by prior removal of FAM134A/C.

The RHD domains of FAM134 family members cluster into highly curved membranes during an early step in ER-phagy initiation, promoting ER membrane budding and scission of ER membrane into autophagosomes for subsequent lysosome degradation.^20,26^ Despite not having obvious LIR motifs to directly bind autophagy machinery, other RHD proteins have propensity for localizing to the curved membranes of ER-phagy buds and could be subsequently co-degraded with the ER-phagy receptors. For example, RHD-containing proteins can associate with FAM134C in co-immunoprecipitation experiments (REEP5 and ARL6P1/5).^26,46^ All RHD proteins (including REEP1/3/4/5/6) accumulated in ATG12^−/−^ iNeurons in a similar way to known ER-phagy receptors, further suggesting autophagic co-clearance of RHD proteins in ER-phagy buds. As further confirmation, we found that Keima-REEP5 clearance was autophagy dependent. However, a more complex pattern was observed upon deletion of FAM134A/C. Some RHD proteins like RTN1-C (and REEP5 to a more limited extent) accumulated both in FAM134A/C^−/−^ and ATG12^−/−^ iNeurons. On the other hand, simply eliminating FAM134 proteins in iNeurons results in a dramatic reduction of REEP1/3/4 abundance (which conversely accumulate in ATG12^−/−^ iNeurons). REEP1–4 have different predicted transmembrane characteristics than REEP5/6 and the N-terminal amphipathic helix of REEP5–6 is not present in REEP1–4 (Fig. 1f)^47^. These differences have been proposed to cause distinct membrane localization and curvature shaping properties for the respective REEP classes^47,48^. The mechanisms underlying loss REEP1/3/4 upon FAM134 deletion are unclear but could reflect co-regulated and/or collaborative functional role in maintaining ER shape. Upstream signals such as phosphorylation are known to activate and possibly cluster multiple ER-phagy receptors^13,49,50^, but further studies are required to understand the relevant regulatory pathways important for axonal ER-phagy bud formation.

ER tubule proteins shape the ER network and facilitate dynamic connections between ER and other organelles, which links each organelle compartment of this highly polarized neuronal cell system to maintain optimal function. Importantly, several proteins found to be controlled via selective autophagy are either linked with human disease (e.g. mutation in ER contact site protein VAPB results in motor neuron disease), or, when experimentally deleted, result in altered ER structure within neurons with functional consequences^5^. This work provides a versatile resource for further interrogating how ER remodeling is optimized for various cell states via selective ER-phagy.

## MATERIALS and METHODS

### Reagents

**Table:** 

REAGENT or RESOURCE	SOURCE	IDENTIFIER	RRID
**Antibodies**			
FAM134B Rabbit Polyclonal Antibody	Proteintech	21537-1-AP	RRID:AB_2878879
FAM134C Rabbit Polyclonal Antibody	Sigma-Aldrich	HPA016492	RRID:AB_1853027
CCPG1 Rabbit Polyclonal Antibody	Cell Signaling Technology	80158	RRID:AB_2935809
TEX264 Rabbit Polyclonal Antibody	Sigma-Aldrich	HPA017739	RRID:AB_1857910
REEP1 Rabbit Polyclonal Antibody	Sigma-Aldrich	HPA058061	RRID:AB_2683591
REEP4 Rabbit Polyclonal Antibody	Sigma-Aldrich	HPA042683	RRID:AB_2571730
REEP5 Rabbit Polyclonal Antibody	Proteintech	14643-1-AP	RRID:AB_2178440
hFAB^™^ Rhodamine Anti-Tubulin Antibody	BioRad	12004166	RRID:AB_2884950
HSP90 mouse monoclonal Antibody	Proteintech	60318	RRID:AB_2881429
Anti-Keima-Red mAb	MBL international	M182-3M	RRID:AB_10794910
Neurofilament heavy polypeptide antibody	Abcam	ab7795	RRID:AB_306084
MAP2 Guinea Pig Polyclonal Antibody	Synaptic systems	188004	RRID:AB_2138181
Nogo-A (C-4) Mouse Monoclonal Antibody	Santa Cruz	sc-271878	RRID:AB_10709573
Calreticulin Rabbit Polyclonal Antibody	Proteintech	10292-1-AP	RRID:AB_513777
GAPDH (D16H11) XP Rabbit Monoclonal Antibody	Cell Signaling Technology	5174	RRID:AB_10622025
Goat anti-mouse Alexa488	Thermo Fisher Scientific	A-11001	RRID:AB_2534069
Goat anti-chicken Alexa488	Thermo Fisher Scientific	A11039	RRID:AB_2534096
Goat anti-rabbit Alexa568	Thermo Fisher Scientific	A-11011	RRID:AB_143157
Goat anti-rabbit Alexa647	Thermo Fisher Scientific	A27040	RRID:AB_2536101
Goat anti-guinea pig Alexa488	Thermo Fisher Scientific	**A-11073**	RRID:AB_2534117
Goat anti-guinea pig Alexa647	Thermo Fisher Scientific	A-21450	RRID:AB_141882
			
			
**Bacterial and virus strains**			
DH5 alpha E. coli competent cells	Homemade		
T1R E. coli Competent cells	Homemade		
**Chemicals, peptides, and recombinant proteins**			
DAPI	Thermo Fisher Scientific	D1306	
TMTpro^™^ 16plex Label Reagent Set	Thermo Scientific	A44520	
Q5 Hot Start High-Fidelity DNA Polymerase	New England BioLabs	M0493	
QuikChange II Site-Directed Mutagenesis Kit	Agilent	200523	
MiSeq Reagent Nano Kit v2 (300 cycles)	Illumina	MS-103-1001	
Bafilomycin A1	Cayman Chemical	88899-55-2	
DAPI (4',6-Diamidino-2-Phenylindole, Dihydrochloride)	Thermo Fisher Scientific	D1306	
16% Paraformaldehyde, Electron-Microscopy Grade	Electron Microscopy Science	15710	
PhosSTOP	Sigma-Aldrich	T10282	
Protease inhibitor cocktail	Roche	4906845001	
TCEP	Gold Biotechnology	TCEP2	
Formic Acid	Sigma-Aldrich	94318	
Trypsin	Promega	V511C	
Lys-C	Wako Chemicals	129-02541	
Urea	Sigma	U5378	
EPPS	Sigma-Aldrich	E9502	
2-Chloroacetamide	Sigma-Aldrich	C0267	
Trypan Blue Stain Thermo Fisher Scientific	Wako Chemicals	129-02541w	
Bio-Rad Protein Assay Dye Reagent Concentrate	Bio-Rad	5000006	
Urea	Sigma	U5378	
EPPS	Sigma-Aldrich	E9502	
2-Chloroacetamide	Sigma-Aldrich	C0267	
Empore SPE Disks C18 3M	Sigma-Aldrich	66883-U	
Pierce Quantitative Colorimetric Peptide Assay	Thermo Fisher Scientific	23275	
GeneArt Precision gRNA Synthesis Kit	Thermo Fisher Scientific	A29377	
12 Well glass bottom plate with high performance #1.5 cover glass	Cellvis	P12-1.5H-N	
Nunc Cell-Culture Nunclon Delta Treated 6-well	Thermo Fisher Scientific	140685	
Nunc Cell-Culture Nunclon Delta Treated 12-well	Thermo Fisher Scientific	150628	
100x21mm Dish, Nunclon Delta	Thermo Fisher Scientific	172931	
Corning Matrigel Matrix, Growth Factor Reduced	Corning	354230	
DMEM/F12	Thermo Fisher Scientific	11330057	
Neurobasal	Thermo Fisher Scientific	21103049	
NEAA	Life Technologies	11140050	
GlutaMax	Life Technologies	35050061	
N-2 Supplement	Thermo Fisher Scientific	17502048	
Neurotrophin-3 (NT-3)	Peprotech	450-03	
Brain-derived neurotrophic factor (BDNF)	Peprotech	450-02	
B27	Thermo Fisher Scientific	17504001	
Y-27632 Dihydrochloride (ROCK inhibitor)	PeproTech	1293823	
Cultrex 3D Culture Matrix Laminin I	R&D Systems	3446-005-01	
Accutase	StemCell	7920	
FGF3	In-house	N/A	
Insulin Human	Sigma-Aldrich	I9278-5ML	
TGF-beta	PeproTech	100-21C	
holo-Transferrin human	Sigma-Aldrich	T0665	
Sodium Bicarbonate	Sigma-Aldrich	S5761-500G	
Sodium selenite	Sigma-Aldrich	S5261-10G	
Doxycycline	Sigma-Aldrich	D9891	
Recombinant SpCas9	Zuris et al., 2015; Orderu		
Hygromycin B	Thermo Fisher Scientific	10687010	
UltraPure 0.5M EDTA, pH 8.0	Thermo Fisher Scientific	15575020	
GlutaMAX	Thermo Fisher Scientific	35050061	
Dulbecco’s MEM (DMEM), high glucose, pyruvate	GIBCO / Invitrogen	11995	
Lipofectamine 3000	Invitrogen	L3000008	
			
**Experimental models: Cell lines**			
HEK293T	ATCC	CRL-1573	CVCL_0045
H9	Wicell	WA9	CVCL_9773
**Recombinant DNA**			
pAC150-Keima-RAMP4	This paper		Addgene 201929
pAC150-Keima-REEP5	This paper		Addgene 201928
pAC150-FAM134C-GFP	This paper		Addgene 201932
pAC150-TEX264-GFP	This paper		Addgene 201931
pAC150-TEX264(deltaLIR, F273A)-GFP	This paper		Addgene 201930
pHAGE-FAM134C-GFP	This paper		Addgene 201927
pHAGE-TEX264-GFP	An et al 2019		Addgene 201925
pHAGE-TEX264(deltaLIR,F273A)-GFP	An et al 2019		Addgene 201926
pHAGE-mCherry-LC3B	An et al 2019		Addgene 201924
**Software and algorithms**			
Prism	GraphPad, V9	https://www.graphpad.com/scientificsoftware/prism/	SCR_002798
SEQUEST	Eng et al., 1994	N/A	
Flowjo	Flowjo, v10.7	https://www.flowjo.com	SCR_008520
Perseus	Perseus v1.6.15.0 Tyanova et al. (2016)	https://maxquant.org/perseus/	SCR_007358
Fiji	ImageJ V.2.0.0	https://imagej.net/software/fiji/	SCR_002285
Imagelab	Biorad, v6.0.1	https://www.bio-rad.com/en-us/product/image-lab-software?ID=KRE6P5E8Z&source_wt=imagelabsoftware_surl	SCR_014210
Cell Profiler	CellProfiler v4.0.6	https://cellprofiler.org/	SCR_007358
Nikon Imaging Software Elements	5.21.3 (Build 1489)		SCR_014329
outknocker.org	http://www.outknocker.org/outknocker2.htm		
ChopChop	https://chopchop.cbu.uib.no/		SCR_015723
**Instruments**			
Orbitrap Fusion Lumos Tribrid Mass Spectrometer	Thermo Fisher Scientific	IQLAAEGAAPFA DBMBHQ	CR_020562
Orbitrap Eclipse Tribrid Mass Spectrometer	Thermo Fisher Scientific	FSN04-10000	SCR_020559
Attune NxT	Thermo Fisher Scientific		SCR_019590
Sony Biotechnology SH800S Cell Sorter	Sony Biotechnology	SH800S	SCR_018066
Neon^™^ Transfection System	Thermo Fisher Scientific	MPK5000	N/A
ChemiDoc MP imaging system	BioRad	12003154	SCR_019037
Yokogawa CSU-X1 spinning disk confocal on a Nikon Ti-E inverted microscope	Yokogawa/ Nikon		

Plasmids constructed for and used in this manuscript will be available at Addgene upon final publication. These include pAC150-Keima-RAMP4 (this paper, Addgene 201929); pAC150-Keima-REEP5 (this paper, Addgene 201928); pAC150-FAM134C-GFP (this paper, Addgene 201932); pAC150-TEX264-GFP (this paper, Addgene 201931); pAC150-TEX264(deltaLIR, F273A)-GFP (this paper, Addgene 201930), pHAGE-FAM134C-GFP (this paper, Addgene 201927); pHAGE-TEX264-GFP (An et al 2019, Addgene 201925); pHAGE-TEX264(deltaLIR,F273A)-GFP (An et al 2019, Addgene 201926); pHAGE-mCherry-LC3B (An et al 2019, Addgene 201924).

The following chemicals, peptides, and recombinant proteins were used: DAPI Thermo Fisher Scientific (D1306); TMTpro^™^ 16plex Label Reagent Set Thermo Scientific (A44520); Q5 Hot Start High-Fidelity DNA Polymerase New England BioLabs (M0493); Gateway LR Clonase II Enzyme Mix Thermo (11791020); NEBuilder HiFi DNA Assembly Master Mix (E2621s); MiSeq Reagent Nano Kit v2 (300 cycles) Illumina (MS-103-1001); Bafilomycin A1 Cayman Chemical (88899-55-2); Sar405 Selective ATP-competitive inhibitor of Vps34 Apexbio (A8883); DAPI (4’,6-Diamidino-2-Phenylindole, Dihydrochloride) Thermo Fisher Scientific (D1306); 16% Paraformaldehyde, Electron-Microscopy Grade Electron Microscopy Science (15710), PhosSTOP Sigma-Aldrich (T10282); Protease inhibitor cocktail Roche (4906845001); TCEP Gold Biotechnology, Formic Acid Sigma-Aldrich (94318); Trypsin Promega (V511C); Lys-C Wako Chemicals (129-02541); Urea Sigma (U5378); EPPS Sigma-Aldrich (E9502); 2-Chloroacetamide Sigma-Aldrich C0267; Trypan Blue Stain Thermo Fisher Scientific Wako Chemicals (129-02541w); Urea Sigma (U5378); EPPS Sigma-Aldrich (E9502); 2-Chloroacetamide Sigma-Aldrich (C0267); Empore SPE Disks C18 3M Sigma-Aldrich (66883-U); GeneArt Precision gRNA Synthesis Kit Thermo Fisher Scientific (A29377); 12 Well glass bottom plate with high performance #1.5 cover glass Cellvis (P12-1.5H-N); Nunc Cell-Culture Nunclon Delta Treated 6-well Thermo Fisher Scientific (140685); Nunc Cell-Culture Nunclon Delta Treated 12-well Thermo Fisher Scientific (150628); 100x21mm Dish, Nunclon Delta Thermo Fisher Scientific (172931); Corning Matrigel Matrix, Growth Factor Reduced Corning (354230); DMEM/F12 Thermo Fisher Scientific (11330057); Neurobasal Thermo Fisher Scientific (21103049); Non Essential Amino Acids Life Technologies (11140050); GlutaMax Life Technologies (35050061); N-2 Supplement Thermo Fisher Scientific (17502048); Neurotrophin-3 (NT-3) Peprotech (450-03); Brain-derived neurotrophic factor (BDNF) Peprotech (450-02); B27 Thermo Fisher Scientific (17504001); Y-27632 Dihydrochloride (ROCK inhibitor) PeproTech (1293823); Cultrex 3D Culture Matrix Laminin I R&D Systems (3446-005-01); Accutase StemCell (7920); FGF3 In-house (N/A); Insulin Human Sigma-Aldrich (I9278-5ML); TGF-beta PeproTech (100-21C); holo-Transferrin human Sigma-Aldrich (T0665); Sodium Bicarbonate Sigma-Aldrich (S5761-500G); Sodium selenite Sigma-Aldrich (S5261-10G); Doxycycline Sigma-Aldrich (D9891); Recombinant SpCas9 Zuris et al., 2015; Hygromycin B Thermo Fisher Scientific (10687010); UltraPure 0.5M EDTA, pH 8.0 Thermo Fisher Scientific (15575020); GlutaMAX Thermo Fisher Scientific (35050061); Dulbecco’s MEM (DMEM), high glucose, pyruvate GIBCO / Invitrogen (11995); Lipofectamine 3000 Invitrogen (L3000008).

### Protocals

Protocols associated with this work can be found on protocols.io with the following DOI: dx.doi.org/10.17504/protocols.io.81wgbx13nlpk/v1

### Cell Culture

Human embryonic stem cells (hESC, H9, WiCell Institute, WA9, RRID: CVCL_9773) were cultured in E8 medium on Matrigel coated plates as described^29^. Cells were split when they reached 80% confluency (every 2–4 days) using 0.5 mM EDTA in 1× DPBS (Thermo Fisher Scientific).

### Neural differentiation of AAVS1-TRE3G-NGN2 pluripotent stem cells

TRE3G-NGN2 was integrated into the AAVS site of the hESCs as previously described (Ordureau et al., 2020). To start differentiation to induced neurons (i-Neurons) from ES cells (day 0), cells were plated at 2x10^5^ cells/ml onto Matrigel coated plates into ND1 medium (DMEM/F12, 1X N2 (thermo), human Brain-derived neurotrophic factor BDNF (Brain derived neurotrophic factor) (10 ng/ml, PeproTech), human Neurotrophin-3 NT3 (10 ng/ml, PeproTech), 1X NEAA (Non-essential amino acids), Human Laminin(0.2 ug/ml) and Doxycycline (2 mg/ml)) also containing Y27632 (rock inhibitor-10 mM)). The media was replaced with ND1 without Y27632 the next day. The following day media was replaced with ND2 (Neurobasal medium, 1X B27, 1X Glutamax, BDNF (10 ng/ml), NT3 (10 ng/ml) and doxycycline at 2 mg/ml. On Days 4 and 6, 50% of the media was changed with fresh ND2. On any day in the Day 4–7 range, cells were replated at 4x10^5^ cells/well in ND2 medium with Y27632. The media was replaced the next day with fresh ND2 (without Y27632) Every other day 50% of the media was changed with ND2. At Day9 and onwards doxycycline was removed from the ND2 mixture. iNeurons were fed every other day with 50% media change until the experimental day (Day12 of differentiation unless otherwise noted).

### Molecular Cloning

Plasmids were made using either Gateway technology (Thermo) or via Gibson assembly (New England biolabs) in pHAGE backbone (for lentivirus transduction) or in the pAC150 piggy Bac backbone (for stable hESC generation). Entry clones from the human orfeome collection version 8 were obtained and cloned via LR cloning into various destination expression vectors.

### Lentivirus generation and Viral transduction of induced neurons

Lentiviral vectors were packaged in HEK293T (ATCC, CRL-1573, RRID: CVCL_0045) by cotransfection of pPAX2, pMD2 and the vector of interest in a 4:2:1 ratio using Lipofectamine 3000. One day after transfection media was changed to ND2 (no DOX) and then the following day virus containing supernatant was collected, filtered through a .22 micron syringe filter and frozen at −80°C. hESCs were differentiated to neurons as described above. At day 11 (two days after Dox removal) the iNeurons were transduced. iNeurons were imaged one day after transduction or at any following day (experimental day noted in each figure).

### Electroporation and selection of stable hESC populations with PiggyBac vectors

PiggyBac plasmids freshly maxi prepped at high concentrations were electroporated into hESCs using the 10μL Neon ThermoFisher kit and ThermoFisher Neon Electroporator. 1.5 μg of pAC150 piggy Bac vectors for ER proteins (Keima-RAMP4, TEX264-GFP, FAM134C-GFP and 1 μg of pCMV-hyPBase hyperactive piggyBac vector. 2x10^5^ cells 10μl buffer R were used for each electroporation. Program 13 was used from the optimization tab for electroporation parameter (Voltage: 1100. Pulse width: 20 Pulse number: 2 ). We plated the electroporated ESCs into Matrigel coated plates containing E8 with Y27632 (rock inhibitor-10 mM) and cells were placed in a low O_2_ incubator for two to four days. After four days with regular E8 media changes daily (or when cells reach 80 percent confluency) the cells were split into selection media (E8 with Y27632 and 50μg/mL hygromycin B). Cell were grown in selection medium for 7–10 days until there was no longer any cell death. Cells were further selected to obtain a fluorophore-positive population via flow cytometry with Sony Biotechnology (SH800S) Cell Sorter.

### Gene Editing

Gene editing in hESCs was performed as in (Ordureau et al., 2018). Guide RNAs (sgRNAs) were generated using the GeneArt Precision gRNA Synthesis Kit (Thermo Fisher Scientific). 0.6 μg sgRNA was incubated with 3 μg SpCas9 protein for 10 min at room temperature and electroporated into 2x10^5^ H9 cells using Neon transfection system (Thermo Fisher Scientific). Cells were put in a low O2 incubator and allowed to recover for 24–72 hours. Cells were then single cell sorted into 96-well plates with Sony Biotechnology (SH800S) Cell Sorter and grown up for 7–12 days. Individual clones were verified for out of frame deletions were verified via DNA sequencing with Illumina MiSeq and protein deletion was verified via immunoblotting. sgRNA target sequences were as follows: CCPG1 sgRNA TTCTAACTTAGGTGGCTCAA, TEX264 sgRNA CATGTCGGACCTGCTACTAC, FAM134A sgRNA TAATACGACTCACTATAG, FAM134B sgRNA GTCTGACACAGACGTCTCAG, FAM134C sgRNA AACTTGAGCTGTCAGACCAACA

### Antibodies

The following antibodies were used: FAM134B Rabbit Polyclonal Antibody Proteintech (21537-1-AP, RRID:AB_2878879); FAM134C Rabbit Polyclonal Antibody Sigma-Aldrich (HPA016492, RRID:AB_1853027); CCPG1 Rabbit Polyclonal Antibody Cell Signaling Technology (80158, RRID:AB_2935809); TEX264 Rabbit Polyclonal Antibody Sigma-Aldrich (HPA017739, RRID:AB_1857910); REEP1 Rabbit Polyclonal Antibody Sigma-Aldrich (HPA058061, RRID:AB_2683591); REEP4 Rabbit Polyclonal Antibody Sigma-Aldrich (HPA042683, RRID:AB_2571730); REEP5 Rabbit Polyclonal Antibody Proteintech (14643-1-AP, RRID:AB_2178440); hFAB^™^ Rhodamine Anti-Tubulin Antibody BioRad (12004166, RRID:AB_2884950); HSP90 mouse monoclonal Antibody Proteintech (60318, RRID:AB_2881429); Anti-Keima-Red mAb MBL international (M182-3M, RRID:AB_10794910); Neurofilament heavy polypeptide antibody Abcam (ab7795, RRID:AB_306084); MAP2 Guinea Pig Polyclonal Antibody Synaptic systems (188004, RRID:AB_2138181); Nogo-A (C-4) Mouse Monoclonal Antibody Santa Cruz (sc-271878, RRID:AB_10709573); Calreticulin Rabbit Polyclonal Antibody Proteintech (10292-1-AP, RRID:AB_513777); GAPDH (D16H11) XP Rabbit Monoclonal Antibody Cell Signaling Technology (5174, RRID:AB_106220250; Goat anti-mouse Alexa488 Thermo Fisher Scientific (A-11001, RRID:AB_2534069); Goat anti-chicken Alexa488 Thermo Fisher Scientific (A11039, RRID:AB_2534096); Goat anti-rabbit Alexa568 Thermo Fisher Scientific (A-11011, RRID:AB_1431570; Goat anti-rabbit Alexa647 Thermo Fisher Scientific (A27040, RRID:AB_2536101); Goat anti-guinea pig Alexa488 Thermo Fisher Scientific (A-11073, RRID:AB_2534117); Goat anti-guinea pig Alexa647 Thermo Fisher Scientific (A-21450, RRID:AB_141882).

### Western-Blotting

Cell pellets were resuspended in 8M Urea buffer (8M Urea, 150 mM TRIS pH, 150 mM NaCl) supplemented with protease and phosphatase inhibitor tablets and then sonicated twice, 10 seconds each, on ice. Lysates were clarified via centrifugation at 20,000 xg for 10 min at 4°C. BCA assays were performed on clarified lysates and normalized lysate amounts were boiled in 1X SDS containing Laemmeli buffer. Lysates were run on 4–20% Tris Glycine gels (BioRad) and transferred via Wet transfer onto PVDF membranes for immunoblotting with the indicated antibodies. Images of blots were acquired using Enhanced-Chemiluminescence or using the Rhodamine channel on a BioRad ChemiDoc imager.

### Flow Cytometry

hESCs that were converting to neurons were grown in 6-well plates and were treated with various drugs for the indicated time points and cell pellets were collected at the indicated day of neuronal differentiation. These were resuspended in FACS buffer (1X PBS, 2% FBS). At least 10,000 cells were analyzed on the Attune NxT (Thermo Fisher Scientific, Cat#A28993)) flow cytometer. Neutral Keima signal was measured at excitation at 445 nm and emission 603 nm with a 48 nm bandpass and acidic Keima signal was measured at 561 nm excitation and emission 620 nm and a 15 nm band pass. The resulting cell population Keima ratio was analyzed as previously described^51^. In brief, FCS files were exported into Flowjo where cells were gated for live cells, single cells and Keima positive cells. The 561(Acidic) to 445 (neutral) excitation ratio was calculated by dividing mean values of 561 nm excited cells to mean values of 445 nm excited cells.

### Imaging

Cells were plated onto 6 well, 12 well or 24 well glass bottom plates with high performance #1.5 cover glass (CellVis). Live cells were imaged at 37°C at 5% CO_2_. For immunofluorescence experiments, cells were fixed at room temperature with 4% paraformaldehyde plus in PBS, solubilized in 0.1% Triton-X in PBS and blocked with 1% BSA/0.1% Triton-X in PBS. Cell were then immunostained with anti-primary antibodies used at 1:500 and then AlexaFluor conjugated antibodies (Thermofisher) used at 1:300. Primary and secondary antibodies used in this study can be found in the Materials Table and described for each experiment detailed below. Fixed cell images were captured at room temperature. Cells were imaged using a Yokogawa CSU-X1 spinning disk confocal on a Nikon Ti-E inverted microscope at the Nikon Imaging Center in Harvard Medical School. Nikon Perfect Focus System was used to maintain cell focus over time. The microscope equipped with a Nikon Plan Apo 40x/1.30 N.A or 100x/1.40 N.A objective lens and 445nm (75mW), 488nm (100mW), 561nm (100mW) & 642nm (100mW) laser lines controlled by AOTF. All images were collected with a Hamamatsu ORCA-Fusion BT sCMOS (6.45 µm^2^ photodiode) with Nikon Elements image acquisition software.

#### Analysis of ER structures in axons.

Cells were fixed and stained as described above specifically with α-Calnexin to detect ER, α-MAP2 to detect dendrites, α-NEFH to mark axons, and DAPI to detect nuclei. Z-stacks were acquired with the parameters stated above. Z series are displayed as maximum *z*-projections and brightness and contrast were adjusted for each image equally and then converted to rgb for publication using FiJi software. Fiji software was also used to split the z projections into individual channels for downstream image analysis in Cell Profiler^52^. Each field of view for all genetic backgrounds was thresholded in the same way with a consistent pipeline. The ‘identify primary objects’ tool was used to find nuclei, axons, dendrites, and ER structures. The α-NEFH-positive axon object regions were used to create an axon mask and ER structures within this mask were counted. The area of each ER structure was also measured. The number of ER axonal structures was then compared to the number of detected nuclei.

#### Visualizing Keima-ER in neuronal differentiation.

Live cells stably expressing Keima-RAMP4 (localizes to all ER) or Keima-REEP5 (localizes to ER tubules specifically) were imaged at the indicated day in neuronal differentiation. Pairs of images for ratiometric imaging of Keima-RAMP4 fluorescence were collected sequentially using 100 mW 442 nm (neutral Keima excitation) and 100 mW 561 nm (acidic Keima excitation) solid state lasers and emission collected with a 620/60 nm filter (Chroma Technologies). Z-stacks were acquired with a Nikon Plan Apo 40×/1.45 N.A oil-objective lens. Z series are displayed as maximum z-projections and brightness and contrast were adjusted for each image equally and then converted to rgb for publication using FiJi software. Fiji software was also used to split the z projections into individual channels. For each channel, complimentary line scans 30 μm long with 1.7 μm width were drawn in either the soma or projection of iNeurons. The 561 nm or 442 nm gray values along these lines was measured using ‘plot profile’ in Fiji. The 561/442 ratio of these values at each complimentary point along the line was calculated and plotted in excel.

#### Characterizing spatial and temporal properties of ER-phagy receptors.

hESCs with WT or ATG12^−/−^ genetic background stably expressing WT or mutant TEX264-GFP or FAM134C-GFP were converted to neurons and treated with various drugs for the indicated time points and imaged at the indicated day in neuronal differentiation. Z-stacks were acquired with the parameters stated above. Z series are displayed as maximum *z*-projections and brightness and contrast were adjusted for each image equally and then converted to rgb for publication using FiJi software.

For day 4 cells (untreated or treated with the indicated drugs), the number of GFP puncta per cell was quantified using Cell Profiler. Each field of view for all genetic backgrounds and drug treatments was thresholded in the same way with a consistent pipeline. Using the ER-phagy receptor (488ex, GFP channel) max z projection image, the ‘identify primary objects’ tool was used to detect cells (receptor labels the whole ER membrane which can be used to identify the cells) and to detect puncta (small bright circles found within the ER membrane). The puncta were linked to each cell and the puncta per cell numbers were exported.

Autophagosome (LC3B) and ER-phagy receptor (TEX264 or FAM134C) co-labelling was achieved by transducing with mCh-LC3B and receptor-GFP lentivirus. Day 30 neurons were imaged live for 30 min with an image acquired every 30 sec. Fiji was used to track GFP and mCh positive puncta. Lines between each frame were used to measure the distance traveled of the puncta from frame to frame. Forward direction was reported as a positive value in micron and backward direction was reported as a negative value. Events in neurons from three independent differentiations were captured. The events were binned based on their speed of movement in micron per second. The percentage of events at each speed were plotted as using GraphPad Prism 7.

After live cell imaging at day 30, the ER-phagy receptor and mch-LC3B positive transduced neurons were fixed as described above. The iNeurons were immunostained with α-MAP2 to detect dendrites and α-NEFH to mark axons. Z-stacks were acquired with the parameters stated above. Z series are displayed as maximum *z*-projections and brightness and contrast were adjusted for each image equally and then converted to rgb for publication using FiJi software.

### Quantitative proteomics

#### Sample preparation for Mass Spectrometry:

Cell pellets were resuspended in 8M Urea buffer (8M Urea, 150 mM TRIS pH, 150mM NaCl) supplemented with protease and phosphatase inhibitor tablets and then sonicated twice, 10 seconds each, on ice. Lysates were clarified via centrifugation at 20,000 xg for 10 min at 4°C. BCA assays were performed on clarified lysates. 100 ug of each sample was taken and total volume raised to 100 μL total. Samples were reduced using TCEP (0.5 M for 30 min at room temperature) and alkylated (with Chloroacetamide (20 mM for 20 min at room temperature) prior to methanol-chloroform precipitation with 3:1 methanol, 1:1 chloroform, and 2.5:1 water added. Aqueous and organic phases were separated with centrifugation for 5 min at 14,000 xg. Liquid around the protein layer was removed and this protein layer was washed with 1 mL methanol and then pelleted at 5 min at 14,000 xg. The supernatant was removed. The pellets were then resuspended in in 50μL, 200 mM EPPS, pH8.5. Peptide digestion was carried out using LysC (1:100) for 2h at 37^o^C followed by Trypsin (1:100) overnight. 25 μL of the digested peptides were then labelled by adding 5 μL 100% acetonitrile (CAN) and with 7 μL of TMT reagent (at 20 mg/ml stock in ACN) for 2h and the reaction was quenched using hydroxylamine at a final concentration of 0.5% (w/v) for 20 min.

#### Basic pH reversed-phase.

Samples were combined 1:1 such that each channel consisted of the same amount of peptide. The pooled peptide sample was desalted with a 100 mg Sep-Pak solid phase extraction column and then fractionated with basic pH reversed-phase (BPRP) HPLC. Fractionation was executed using an Agilent 1200 pump with an Agilent 300 Extend C18 column (3.5 μm particles, 2.1 mm ID, and 250 mm in length). A 50 min linear gradient from 5% to 35% acetonitrile in 10 mM ammonium bicarbonate pH 8 at a column flow rate of 0.25 mL/min was used for peptide fractionation. A total of 96 fractions were collected and then concatenated down to 24 superfractions, as described previously ^53^. These 24 superfractions were divided into two sets of 12 non-adjacent superfractions and were acidified by adding formic acid to a concentration of 1%. One set of fractions (n=12) were vacuum centrifuged to near dryness, and each was desalted via StageTip, dried by vacuum centrifugation, and reconstituted in 5% acetonitrile, 5% formic acid prior to LC-MS/MS analysis.

Mass spectrometry data acquisition and processing. Mass spectrometric data were collected on an Orbitrap Fusion Lumos mass spectrometer coupled to a Proxeon NanoLC-1200 UHPLC and a FAIMSpro interface ^54^. The 100 μm capillary column was pulled in-lab and packed with 35 cm of Accucore 150 resin (2.6 μm, 150 Å; ThermoFisher Scientific). Peptides were eluted over a gradient of 90 or 110 min. consisting of 5% acetonitrile to 30% acetonitrile in 0.125% formic acid. The scan sequence began with an MS1 spectrum (Orbitrap analysis, resolution 60,000, scan range 350–1350 or 400–1600 Th, automatic gain control (AGC) target was set as “standard,” maximum injection time was set to auto). SPS-MS3 analysis was used to reduce ion interference ^55,56^. MS2 analysis consisted of collision-induced dissociation (CID) and quadrupole ion trap analysis (automatic gain control (AGC) 2 x10^4^, normalized collision energy (NCE) 35, q-value 0.25, maximum injection time 35 ms, isolation window 0.7 Th). Following the acquisition of each MS2 spectrum, we collected an MS3 spectrum in which multiple MS2 fragment ions were captured in the MS3 precursor population using an isolation waveform with multiple frequency notches. MS3 precursors were fragmented by higher-energy collisional dissociation (HCD) and analyzed using the Orbitrap (NCE 55, AGC 1.5 x10^5^, maximum injection time 150 ms, resolution 50,000). We used the Real Time Search (RTS) using Orbiter^57^ with a *H. sapiens* database (UniProt, downloaded August 2020) and we limited MS3 scans to 2 peptides per protein per fraction. A total of 24 RAW files were collected with data for 12 non-adjacent superfractions acquired using a compensation voltage (CV) set of −40/−60/−80V with a 1.25-sec TopSpeed cycle was used for each CV.

Spectra were converted to mzXML via MSconvert ^58^. Database searching included all *H. sapiens* entries from UniProt. The database was concatenated with one composed of all protein sequences in that database in the reversed order. Searches were performed using a 50-ppm precursor ion tolerance for total protein level profiling. The product ion tolerance was set to 0.9 Da. These wide mass tolerance windows were selected to maximize sensitivity in conjunction with SEQUEST ^59^ searches and linear discriminant analysis ^60,61^. TMT labels on lysine residues and peptide N termini (+304.207 Da) and carbamidomethylation of cysteine residues (+57.021 Da) were set as static modifications, while oxidation of methionine residues (+15.995 Da) was set as a variable modification. Peptide-spectrum matches (PSMs) were adjusted to a 2% false discovery rate (FDR) ^62,63^. PSM filtering was performed using a linear discriminant analysis, also as described previously ^61^ and then assembled further to a final protein-level FDR of 2% ^63^.

Proteomics Data analysis. Peptide-spectral matches (PSM) were filtered for summed signal-to-noise ratio (SNR>200) across the TMT plex and for precursor signals that contained > 0.5 isolation purity of the MS1 isolation window. To normalize protein input across TMT channels, all PSM intensities were summed and the total intensity per channel were sum normalized to the median summed intensity across the TMTpro plex. Protein intensities were generated by summing input-normalized TMT intensities for its constituent peptides’ PSMs^64^, serving as a weighted average quantification. Comparison among experimental conditions (n=3–4 biological replicates) were conducted by performing a Student’s t-test of normalized log_2_-transformed protein TMT intensities. Resulting p-values were adjusted for multiple hypothesis correction using the Benjamini-Hochberg approach^65^. For heatmap generation or linear model analysis, replicate protein report ion intensities were normalized to the mean of the biological replicates of either day0 for the differentiation experiment or to wildtype control day12 iNeurons replicates within a given TMTpro plex.

To conduct the linear regression analysis using a single model for the additive combinatorial ER receptor knockout TMT data, we incorporated indicators/dummy variables that can take on one of two possible numerical values (1: contains addition of an ER receptor knockout(s) or 0: does not). All replicates were normalized to mean of WT control which was centered at 0, essentially removing the intercept estimation (β_0_) from the model. This was because the TMT protein reporter intensities are not indicative of absolute abundance, and we are interested in understanding the fold change contribution from the addition of each ER receptor knockout. The following indicators/dummy variables were used:

$$x_{i1}=\left\{\begin{array}{ll} 1 & \text{if }i\text{th sample contains FAM}134{\text{A}}^{-/-}/{\text{C}}^{-/-}\\ 0 & \text{if }i\text{th sample does not contain FAM}134{\text{A}}^{-/-}/{\text{C}}^{-/-} \end{array}\right.$$

$$x_{i2}=\left\{\begin{array}{rr} 1 & \text{if }i\text{th sample contains FAM}134{\text{A}}^{-/-}/{\text{B}}^{-/-}/{\text{C}}^{-/-}\\ 0 & \text{if }i\text{th sample does not contain FAM134}{\text{A}}^{-/-}/{\text{B}}^{-/-}/{\text{C}}^{-/-} \end{array}\right.$$

$$x_{i3}=\left\{\begin{array}{rr} 1 & \text{if }i\text{th sample contains FAM134}{\text{A}}^{-/-}/{\text{B}}^{-/-}/{\text{C}}^{-/-}/\text{TEX}264^{-/-}\\ 0 & \text{if }i\text{th sample does not contain FAM134}{\text{A}}^{-/-}/{\text{B}}^{-/-}/{\text{C}}^{-/-}/\text{TEX}264^{-/-} \end{array}\right.$$

$$x_{i4}=\left\{\begin{array}{rr} 1 & \text{if }i\text{th sample contains FAM134}{\text{A}}^{-/-}/{\text{B}}^{-/-}/{\text{C}}^{-/-}/\text{TEX}264^{-/-}/\text{CCPG}1^{-/-}\\ 0 & \text{if }i\text{th sample does not contain FAM134}{\text{A}}^{-/-}/{\text{B}}^{-/-}/{\text{C}}^{-/-}/\text{TEX}264^{-/-}/\text{CCPG}1^{-/-} \end{array}\right.$$

The model below was then used to estimate betas (β) for each step-wise addition of ER receptor knockout(s) with inherent technical noise from the MS acquisition and reporter quantification.


$$Y={\text{β}}_{0}+{\text{β}}_{WT\to DKO}x_{i1}+{\text{β}}_{DKO\to TKO}x_{i2}+{\text{β}}_{TKO\to QKO}x_{i3}+{\text{β}}_{QKO\to PKO}x_{i4}+\epsilon_{i}$$

Thus:

$${\text{β}}_{0}+\epsilon_{i}\to \text{if }i\text{th sample is WT}$$

$${\text{β}}_{0}+{\text{β}}_{WT\to DKO}x_{i1}+\epsilon_{i}\to \text{if }i\text{th sample is a FAM134}{\text{A}}^{-/-}/{\text{C}}^{-/-}\text{ KO}$$

$${\text{β}}_{0}+{\text{β}}_{WT\to DKO}x_{i1}+{\text{β}}_{DKO\to TKO}x_{i2}+\epsilon_{i}\to \text{if }i\text{th sample is a FAM134}{\text{A}}^{-/-}/{\text{B}}^{-/-}/{\text{C}}^{-/-}\text{ KO}$$

$$\begin{array}{l} {\text{β}}_{0}+{\text{β}}_{WT\to DKO}x_{i1}+{\text{β}}_{DKO\to TKO}x_{i2}+{\text{β}}_{TKO\to QKO}x_{i3}+\epsilon_{i}\\ \;\to \text{if }i\text{th sample is a FAM134}{\text{A}}^{-/-}/{\text{B}}^{-/-}/{\text{C}}^{-/-}/\text{TEX}264^{-/-}\text{ KO} \end{array}$$

$$\begin{array}{l} {\text{β}}_{0}+{\text{β}}_{WT\to DKO}x_{i1}+{\text{β}}_{DKO\to TKO}x_{i2}+{\text{β}}_{TKO\to QKO}x_{i3}+{\text{β}}_{QKO\to PKO}x_{i4}+\epsilon_{i}\\ \;\to \text{if }i\text{th sample is a FAM134}{\text{A}}^{-/-}/{\text{B}}^{-/-}/{\text{C}}^{-/-}/\text{TEX}264^{-/-}/{\text{CCPG}}^{-/-}\text{ KO} \end{array}$$

In R using the *lm* function, the beta (β) coefficients and p-values were extracted from the model, and beta (β) coefficients and Benajmini-Hochberg^65^ adjusted p-values (q-values) were leveraged for downstream analysis and figure generation. One can interpret the β_TKO→QKO_ for instance as the average foldchange from the triple knockout to the quadruple knockout, due to the addition of TEX264 KO on the FAM134A^−/−^/B^−/−^/C^−/−^ knockout cells.

Classification of proteins to various organellar locations or functional groups were performed using manually curated databases from Uniprot and are listed in the relevant supplementary tables. Sub-cellular annotations were derived from Itzak et al.^30^ with additional cytosol protein location designations from Uniprot. ER high sheet and high curvature annotations were extracted from ref^10^.

### Proteomics Data Availability

The mass spectrometry proteomics data have been deposited to the ProteomeXchange Consortium via the PRIDEpartner repository^66^ with the dataset identifier PXD041069 and reviewers can access it.

### Code Availability

Code for proteomics data analysis and relevant figure generation can be found on GitHub at the https://github.com/harperlaboratory/iNeuron_ERphagy.git repository.

### Statistics

Proteomics data analysis was performed using R (4.2.2) within the Rstudio IDE (2022.12.0 Build 353, Posit). Data visualizations in the form of heatmaps, volcano plots, violin plots, protein abundance profiles, and subcellular localization plots were generated using the following R packages: tidyverse (2.0.0), dplyr (1.0.10), cowplot (1.1.1), pheatmap (1.0.12), stringr (1.5.0), RColorBrewer (1.1–3), ggrepel (0.9.2), ggplot2 (3.4.1), purr (1.0.1), and tibble (3.1.8). For imaging statistics, GraphPad Prism9 was used. Mean (for number of ER structures per nuclei) or median (for the area of axonal ER structures) values from each replicate differentiation experiment (n=4) were compared between each knockout and wildtype using a paired t test assuming a Gaussian distribution. For flow cytometry quantification, GraphPad Prism9 was used. Each condition had three biological replicates. Brown-Forsythe and Welch One-way ANOVA and Dunnett’s T3 multiple comparisons test (assuming a Gaussian distribution) were used to compare each condition. For imaging and flow cytometry analysis *, p<0.05; **, p<0.01, ***, p<0.001. For proteomics datasets, the alpha used for FDR cut-offs was q-values <0.05 to consider significance. All data figures were generated in Adobe Illustrator using R (4.1.3), Rstudio IDE(2021.09.3 Build 396, Posit), and GraphPad Prism9. Unless stated otherwise all quantitative experiments were performed in triplicate and average with S.E.M. or S.D. as indicated in legends reported.

## Extended Data

## Supplementary Material

Supplement 1

Supplement 2

Supplement 3

Supplement 4

Supplement 5

Supplement 6

Supplement 7 **Supplemental Movie 1, related to** Fig. 2. An additional example of day 30 WT iNeurons expressing TEX264-GFP (green) and mCh-LC3B (red) imaged every 30s. Image sequence played at 2 frames per second. Circles indicate co-trafficking of TEX264-GFP/mCh-LC3B positive puncta. Scale bar 10 microns.
**Supplemental Movie 1, related to** Fig. 2. Day 30 WT iNeurons expressing FAM134C-GFP (green) and mCh-LC3B (red) imaged every 30s. This example movie includes the cropped region represented in Fig. 2j. Image sequence played at 2 frames per second. Circles indicate co-trafficking FAM134C-GFP/mCh-LC3B positive puncta. Scale bar 10 microns.

Supplement 8

Supplement 9 **Supplemental Movie 3, related to** Fig. 2. A region of a day 30 WT iNeuron expressing TEX264-GFP (green) and mCh-LC3B (red) imaged every 20s. This example movie is the region represented in Fig. 2k where a TEX264-GFP/mCh-LC3B positive puncta leaves the dilated axonal region. Image sequence played at 2 frames per second. Scale bar 5 microns.
